# Spatial analysis of nanoparticle distribution in human breast xenografts reveals nanoparticles targeted to cancer cells localized with tumor-associated stromal cells

**DOI:** 10.7150/ntno.84255

**Published:** 2023-06-26

**Authors:** Sean Healy, Elizabeth T Henderson, Suqi Ke, Jacqueline Kelly, Brian W Simons, Chen Hu, Robert Ivkov, Preethi Korangath

**Affiliations:** 1 Department of Biomedical Engineering, Whiting School of Engineering, Johns Hopkins University, Baltimore 21218, USA.; 2 Dept of Radiation Oncology and Molecular Radiation Sciences, School of Medicine, Johns Hopkins University, Baltimore, MD 21231 USA.; 3 Department of Biostatistics and Bioinformatics, Sidney Kimmel Comprehensive Cancer Centre, School of Medicine, Johns Hopkins University, Baltimore, MD 21231 USA.; 4 Center for Comparative Medicine, Baylor College of Medicine, Houston, TX USA.; 5 Dept of Oncology, Sydney Kimmel Comprehensive Cancer Centre, School of Medicine, Johns Hopkins University, Baltimore, MD 21231 USA.; 6 Department of Mechanical Engineering, Whiting School of Engineering, Johns Hopkins University, Baltimore 21218, USA.; 7 Department of Materials Science and Engineering, Whiting School of Engineering, Johns Hopkins University, Baltimore 21218, USA.

**Keywords:** Immune response, nanoparticles, spatial analysis, stromal cells, targeted delivery.

## Abstract

The biological influence of physicochemical parameters of “targeted” nanoparticles on their delivery to cancer tumors remains poorly understood. A comparative analysis of nanoparticle distributions in tumors following systemic delivery across several models can provide valuable insights.

**Methods:** Bionized nanoferrite nanoparticles (iron oxide core coated with starch), either conjugated with a targeted anti-HER2 antibody (BH), or unconjugated (BP), were intravenously injected into athymic nude or NOD-scid gamma (NSG) female mice bearing one of five human breast cancer tumor xenografts growing in a mammary fat pad. Tumors were harvested 24 hours after nanoparticle injection, fixed, mounted, and stained. We performed detailed histopathology analysis by comparing spatial distributions of nanoparticles (Prussian blue) with various stromal cells (CD31, SMA, F4/80, CD11c, etc.) and the target antigen-expressing (HER2) tumor cells.

**Results:** Only BH nanoparticles were retained in tumors and generally concentrated in the tumor periphery, with nanoparticle content diminishing towards the tumor interior. Nanoparticle distribution correlated strongly with specific stromal cells within each tumor type, which varied among tumor types and between mouse strains. Weak or no correlation between nanoparticle distribution and HER2 positive cells, or CD31 cells was observed.

**Conclusion:** Antibody-labeled nanoparticles were retained across all tumors, irrespective of presence of the “target” antigen. Though presence of antibody on nanoparticles correlated with retention, non-cancerous host stromal cells were responsible for their retention in the tumor microenvironment. This study highlights gaps in our understanding of the complex biological interplay between disease and host immune biology, and the need to account for the influence of underlying aberrant tumor biology as factors determining nanoparticle fate in vivo.

## Introduction

Engineered nanoparticles display immense potential for diagnosis and therapy of various diseases including cancer 1-4. The most critical factor and greatest challenge in developing nanoparticles for cancer therapy is ensuring their reliable delivery into solid tumors. Nanomedicine development continues to focus largely towards improving passive delivery of nanoparticles to solid tumors by changing their physical and chemical characteristics 5-9. Demonstrations of successful nanoparticle delivery remains limited, with only a handful of nanoparticle-based cancer therapeutics approved for clinical use. In these cases, approval of cancer nanomedicine formulations often resulted from nanoparticle-altered drug bio distribution and toxicity, compared to free drug, rather than increasing concentration of drug in tumor 10-11. Furthermore, a meta-analysis of free doxorubin versus liposomal doxorubicin administered to patients showed no difference between the two cohorts in terms of objective response 12. Successful clinical translation of a “targeted” nanoparticle, i.e. nanoparticle conjugated with an “active” targeting moiety towards tumor cells, remains an unrealized goal. There is a growing recognition that a barrier to achieving this ideal is our lack of a clear understanding of biological mechanisms leading to nanoparticle uptake and distribution in solid tumors.

Enhanced permeability and retention (EPR) arising from aberrant and leaky vasculature in the tumor remains the accepted mechanism for nanoparticle access and retention in the tumor microenvironment. “Passive” retention is thought to be dominated by nanoparticle size slowing reentry into blood 13-14. Using this rationale, increased bioavailability through prolonged blood circulation enhances passive nanoparticle access and entry into the tumor, motivating development of stealth nanoparticles exhibiting increased circulation time 15-17. Original observations supporting the development of the EPR hypothesis were from human tumors grown in immunocompromised mouse models.

For the past several decades, preclinical studies in cancer biology and drug development have relied heavily on immunocompromised mouse models. These models have utility in cancer drug development for human disease because they provide a permissive environment for tumor grafts to grow from human cancer cells, enabling efficient drug screening, and providing useful insights into gene- or pathway-specific therapy strategies. Small-molecule anti-cancer drugs development has been enhanced by use of these models because they provide a complete, living system for testing. More recently, a growing recognition that the host immune system plays a pivotal role in both disease progression and response to therapy has motivated calls for careful evaluation of such strategies, particularly for nanoparticle-based formulations (11, 18, 19).

Nanoparticles, by virtue of their size and other physiochemical properties, interact strongly with the host immune system, altering their fate in vivo and adding to the biological complexities of host and disease. Hence, there is increasing acknowledgement that interpretation of results obtained from pre-clinical studies relying exclusively on immunocompromised models is complex, as these models fail to recapitulate clinical realities. The original optimism fueled by early preclinical successes were not recapitulated in clinical trials, motivating the recent reevaluation of development strategies in the context of a perceived disconnect between expectations and realities of clinical translation 18, 19. For example, the first and most successful nanoparticle-based cancer drug, Doxil^®^, achieved approval on its reduced toxicity profile compared to free drug rather than on its ability to raise intratumor levels of chemotherapy or its superior efficacy 20.

A more recent survey of data from mouse models, suggests that on average, the amount of nanoparticle retained in the tumor is about 0.7% of the injected dose 21. Furthermore, studies using improved visualization techniques across expanded models provide limited support for EPR as variable biology among tumor types, and individual variations confound a universal “one-size-fits-all” approach 22-24. As shown by Sindhwani et al 24, gold nanoparticle transport to solid tumors occurs through active uptake by endothelial cells. This study highlights the role of active transport for nanoparticle uptake by non-cancer cells in the tumor microenvironment.

Taken together, these and other studies point towards unanticipated complexities in our understanding of nanoparticle pharmacodynamics, with a growing realization that we must implement a nuanced and careful approach to our use and interpretation of data obtained from animal models, in order to improve our understanding of the relevance of these results to clinical translation. Though immune deficient mouse models may fail to represent faithfully all complexities encountered in the clinical setting, they remain a superior class of living models to study effects of diverse tumor and host biology on nanoparticle delivery 25-26. Further, these models provide a means to ascertain potential effectiveness experimental pharmaceuticals for treating human diseases in varied biological contexts, an important consideration for early stage preclinical development. For cancer nanomedicine specifically, these models enable comparative studies exploring strategies to achieve “active” or “targeted” delivery, e.g. anti-HER2 antibody, specific to a feature of tumor biology, e.g. HER2 status, in different immunologic contexts. Such studies are critical, given reports of substantial discrepancies between results obtained from cell culture and animal models. From these models, in depth and microscopic analysis comparing cellular distribution with nanoparticle distributions in tumors can offer valuable insights into nanoparticle interactions with host cells. Such information can prove valuable to understand biological effects important for developing nanoparticles for theranostic applications, particularly with regard to understanding the influence that biology of the chosen model exerts on the outcome. To be effective, and avoid misleading conclusions, interpretation of results from immune compromised models must explicitly consider the considerable limitations for direct clinical translation.

In our previous study, we observed that the immune status of the host, rather than antigen-antibody interactions with cancer cell membranes dominated retention of antibody-conjugated nanoparticles in tumor models. Flow cytometry analysis of nanoparticle-associated cells revealed less interaction between HER2 positive tumor cells and Herceptin^®^ (monoclonal antibody towards human epidermal growth factor receptor 2 (HER2) protein) conjugated nanoparticles 26, contrary to in vitro results, despite their higher retention in tumors when compared against their plain unconjugated counterpart.

Here, we revisit those initial findings with a detailed histopathology quantitation of the spatial distribution of Herceptin^®^-conjugated iron oxide nanoparticles in tumors obtained from the five (human-mouse xenograft) breast cancer models used previously. The spatial distribution of the nanoparticles was then compared to that of blood vessels present in the tumor identified by endothelial cell marker CD31, proinflammatory and dendritic cells using CD11c, tumor cells with HER2 staining, macrophages identified using F4/80, fibroblasts using alpha smooth muscle actin (SMA) and collagen by Masson's Trichrome staining. We found that the nanoparticles predominantly accumulated on the outer quartile of the tumors, irrespective of tumor type (HER2 status) or mouse background. Additionally, the distribution pattern correlated better with intratumor stromal cell distributions than with HER2 expressing tumor cells across all tumor models. In each tumor type however, nanoparticle distributions correlated better with one or more type of stromal cells that differed among the models.

## Methods

All tissues analyzed in this study were collected as a part of our previously published work 26. The Institutional Animal Care and Use Committee (IACUC) at Johns Hopkins University approved those studies.

Briefly, five different breast cancer cell lines of varying human epidermal growth factor receptor 2 (HER2) protein (HER2 positive cell lines: MCF7/HER, HCC1954, and BT474; and HER2 negative cell lines: MDA-MB-231 and MCF7/Neo) were orthotopically injected into the 4^th^ mammary gland on both sides of either athymic nude (BALB/c) or NOD-SCID gamma (NSG) mice. When the tumor volume reached approximately 150mm^3^ measured with calipers, mice were randomly assigned to intravenously receive phosphate buffered saline (PBS), Bionized nano ferrite plain (BP), or Herceptin^®^-conjugated BNF (BH) nanoparticles (5mg Fe/mouse). After 24hrs, all mice were euthanized and tumors were collected. One-half of each tumor was fixed in 10% formalin and embedded in paraffin and sectioned for histological staining. Prussian blue staining was conducted on all slides to evaluate the distribution of iron nanoparticles. Immunohistochemistry analyses for endothelial cell marker CD31, target tumor antigen HER2, macrophage marker F4/80, fibroblast marker alpha- smooth muscle actin (a-SMA), dendritic and other proinflammatory cell marker CD11c, were performed on tumors from PBS treated mice. Masson's trichrome and Prussian blue staining were done on BH treated tumor slides through Johns Hopkins University reference histology core. All stained slides were then scanned with an Aperio ScanScope At or CS system (Aperio, Vista, CA) at 40× magnification. Tissue that were folded or damaged after staining were excluded from the analysis. Total number of tumors analyzed from each group for each stain are given in Table S1. Digitized images (in .svs format) were accessed using FIJI/ImageJ software.

### Immunohistochemistry

Detailed staining procedures for CD31 and HER2 have been described previously 26. Slides from that study were used for Prussian blue, CD31 and HER2 analysis. Immunostaining for F4/80 was performed on formalin‐fixed, paraffin embedded sections using a Ventana Discovery Ultra autostainer (Roche Diagnostics, Indianapolis, IN) at the Oncology Tissue Services Core of Johns Hopkins University.

Briefly, following dewaxing and rehydration on board, slides were soaked in Target Retrieval Solution (Dako/Agilent - S170084-2, Santa Clara, CA) for 48 minutes at 96 ºC for antigen retrieval. Primary antibody, anti‐ F4/80 (1:2000 - Serotec, now Biorad MCA497, Hercules, CA) was applied at 36ºC for 60 minutes, followed by rabbit anti-rat linker antibody (1:500 - Vector Labs AI4001, Newark, CA) at 36ºC for 32 minutes. Linker antibodies were detected using an anti-rabbit HQ detection system (Roche Diagnostics - 7017936001 and 7017812001, Indianapolis, IN) followed by Chromomap DAB IHC detection kit (Roche Diagnostics-5266645001, Indianapolis, IN), counterstaining with Mayer's hematoxylin, dehydration and mounting.

Immunostaining of CD11c and α-SMA were performed as follows. After deparaffinization and rehydration with successive soaking in 100%, 95%, and 70% ethanol/water, all slides were washed in deionized water followed by dipping in 0.1% Tween and citrate buffer. Antigen retrieval was performed for CD11c slides in citrate buffer by heating in a steamer for 45 minutes. No antigen retrieval was needed for α-SMA staining. After cooling slides were washed with PBS-T and blocked with peroxidase blocking solution (Agilent - S200389-2, Santa Clara, CA) for CD11c staining and 30 min blocking with 0.5% BSA for α-SMA staining. Primary antibodies (CD11c (1:100 - Cell Signaling 97585S, Danvers, MA); α-SMA (1:200 - Abcam ab5694, Waltham, MA) were then added and samples incubated at room temperature for 45 minutes. Following a wash in TBS-T, Poly-HRP anti-Rabbit IgG (Power Vision poly HRP anti rabbit (PV6119), Leica Biosystems, Deer Park, IL) secondary antibody was added and slides were incubated at room temperature for 30 min. Following a thorough wash, DAB (Sigma Fast DAB tablets - D4293-50SET, MilliporeSigma, Burlington, MA) was used as chromogen and slides were counterstained with Mayer's hematoxylin (Agilent -S330930-2 Santa Clara, CA) before dehydration and mounting.

### Image Acquisition and Adjustments

FIJI/ImageJ (version 1.51n, National Institutes of Health, Bethesda, MD, USA) software was used to visualize and characterize nanoparticle distribution patterns in Prussian blue and immunohistochemistry (IHC) stained tumor cross-sections.

Digitized images of stained tumors were imported into ImageJ using the BioFormats plugin. Images were then transformed into RGB color space, color thresholded to select for blue pixels (representative of the nanoparticles), and then binarized. Color threshold parameters were optimized as follows:

Prussian blue: Hue range: 123-196; Saturation range: 0-255; Brightness range: 0-222

Imported stained tumor images were then processed to set threshold values to select for IHC stained pixels. The color threshold parameters for IHC stained tumors were as follows:

IHC: Hue range: 0-25; Saturation range: 0-255; Brightness range: 0-150

In particular, HER2 stained tumors possessed non-negligible and uniformly distributed background staining relative to other IHC stains. To address, additional processing was performed using the Color Deconvolution tool, which unmixes an RGB image produced by subtractive mixing into separate channels corresponding to one to three determined colors. For HER2 stained tumors, the H DAB vector was used, after which a threshold value of 110 was used to reduce background.

Finally, imported stained tumor images were processed to select threshold values to select for positive pixels following the Masson's Trichrome staining procedure. The color threshold parameters for Masson's Trichrome stained tumors were as follows:

Masson's trichrome: Hue range: 123-235; Saturation range: 0-255; Brightness range: 0-180

### Constructing Regions of Interest (ROI) with the FIJI “ROI Manager”

The tumor boundary was defined by a user-drawn region of interest (ROI), and then partitioned into four bands, or quartiles, of equal area. This process was carried out for all slides using ImageJ in the following manner: First, an ROI was drawn to outline the tumor boundary (with a 50 μm extension beyond the outer edge of the stained tumor to ensure that the outermost edges of the tumor were included). This outline (the primary ROI) was duplicated and reduced in size to enclose a smaller segment that comprised exactly 75% of the total tumor area. The area between these two lines/ROIs formed the first quartile, which comprised the outer 25% of the tumor area. Next, a second ROI was duplicated and reduced to form a third ROI that enclosed 50% of the total area. The area between the second and third ROIs formed the second quartile (which had the same total area as the first quartile). This process was repeated iteratively until a total of four quartiles, each encompassing 25% of the total tumor area, was defined.

The “Enlarge” tool in ImageJ, which enables objects to be rescaled, was used to generate the nested ROIs. The “XOR” tool (i.e. “exclusive OR,” which calculates the inverse of the intersection between two shapes) was then used to calculate the area of each quartile by comparing pairs of adjacent ROIs to measure the region between. For example, when the first and second ROIs were selected, the XOR tool finds the overlapping area between them and then takes the inverse, which represents the area of the first quartile.

### Data Analysis

The pixel density - the number of positive stained pixels divided by the number of all pixels within a tumor region - was calculated for each ROI using the ImageJ “Analyze Particles” tool. After evaluating pixel densities in each quartile, differences in nanoparticle distribution in tumors among tumor models were represented graphically. Necrotic regions, artifacts, and any suspicious areas of background staining within the tumor were removed from the initial user drawn ROI and subsequent analysis. Positive pixel density within each quartile was normalized by total positive pixel count (100%) within the respective tumor to compare positive pixel density over each of the four quartiles, allowing for comparisons in positive pixel distribution among nanoparticle and cell type distribution.

### Statistical analysis

All quartile datasets were analyzed using nonparametric Mann-Whitney test using Graphpad Prism software. Coefficient of determination (R^2^) was used to quantify the correlation of normalized pixel positivity of each quartile to total positive pixel count between different stains. R^2^ ranges between 0 and 1 where a higher value indicates a stronger correlation. In order to account for the unbalanced sample sizes encountered among the different groups representing individual stains, and to quantify the variability when estimating R^2^, a resampling-based approach was used. Specifically, within each quartile of each cell type, staining results were resampled with replacement to have a sample size of 100 and the corresponding sample median within each quartile was recorded. R-square of these sample medians between nanoparticles (PB) and each cell type (represented by stain-group) was calculated. By repeating the bootstrap resampling for 1,000 times, we recorded the 2.5^th^, 50^th^, and 97.5^th^ percentile of the resulting 1,000 R^2^ and these were reported. Given the study nature and small sample size, we consider the correlation is strong if both 2.5^th^ of R^2^ was greater than 0.8, and 50^th^ percentile of R^2^ was greater than 0.9.

## Results

**Figure 1:**
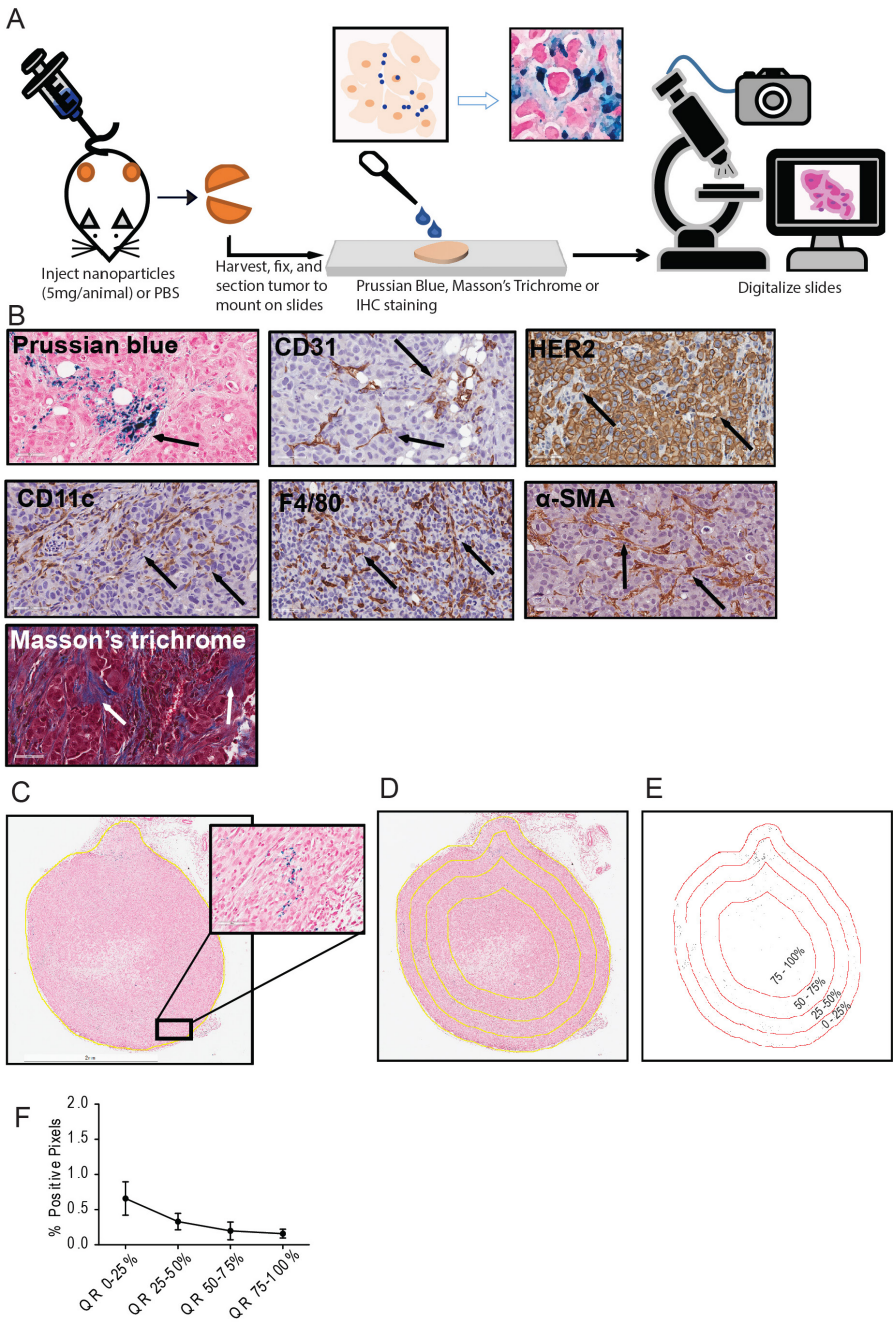
** Schema of the study and analysis**. A. Five breast cancer tumor cell lines - MDA-MB-231, MCF7/NEO, MCF7/HER, BT474, and HCC1954 - were grown in both athymic nude (nude) and NOD-Scid gamma (NSG) mice. When the tumor volume reached ~150mm3, animals were injected with PBS, BNF-Plain (BP) or BNF-HER (BH) nanoparticles (5mgFe/animal). After 24 hours, tumors were harvested and bisected. One half was fixed in formalin and cross-sectioned for histological analysis. Tissue slides were stained for identification of iron nanoparticles by Prussian blue (PB), different protein markers for cell types by immunohistochemistry and for collagen (Masson's trichrome). All stained slides were digitalized in order to computationally evaluate nanoparticle and different types of cell distribution. B. Representative images for each type of staining - Prussian blue (PB) for iron, CD31 for blood vessels, CD11c for proinflammatory cells and dendritic cells, HER2 for tumor cells, F4/80 for macrophages, alpha smooth muscle actin (α-SMA) for fibroblasts and Masson's trichrome for collagen. C. Representative image of a digitalized PB stained tumor. Image was imported into ImageJ/Fiji, and then annotated to form a Region of Interest (ROI) defining the tumor boundary (non-tumor tissue and necrotic regions were excluded from analysis). Cells that stained positively for iron appear blue, as shown in the magnified selection. D. Each tumor is divided into 4 quartiles of equal areas with ROI. E. The image was then processed in binary and analyzed to count the number of positive pixels. F. The positive pixel density quantitated from each quartile (QR- quartile region) were used to generate resulting graphs.

**Figure 2:**
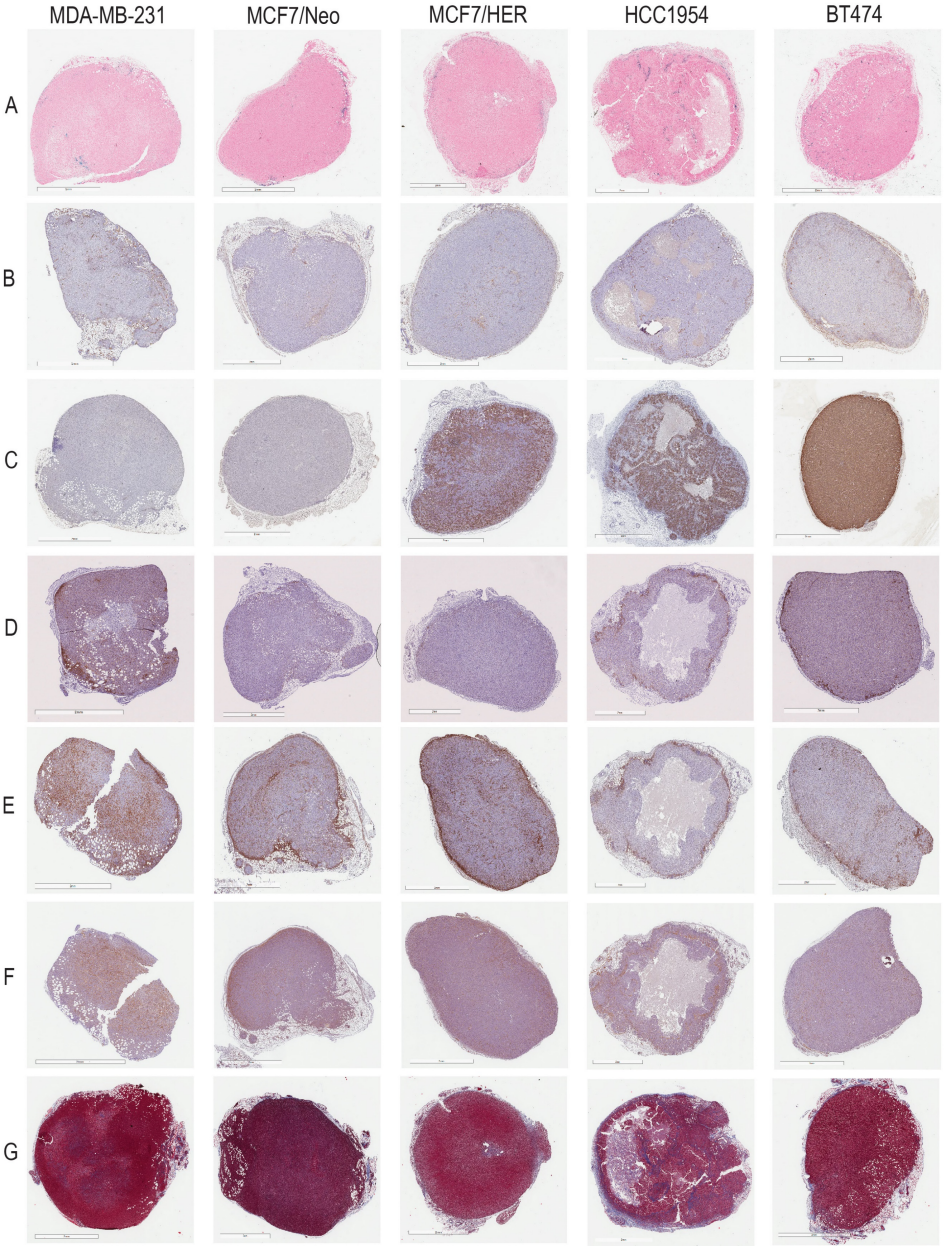
** Representative images for each staining in nude xenografts.** A. Prussian blue staining for nanoparticles. B. CD31 for blood vessels. C. HER2 for tumor cells. D. CD11c for dendritic cells and other proinflammatory cells. E. F4/80 for macrophages. F. SMA for fibroblasts. G. Masson's Trichrome for collagen.

**Figure 3:**
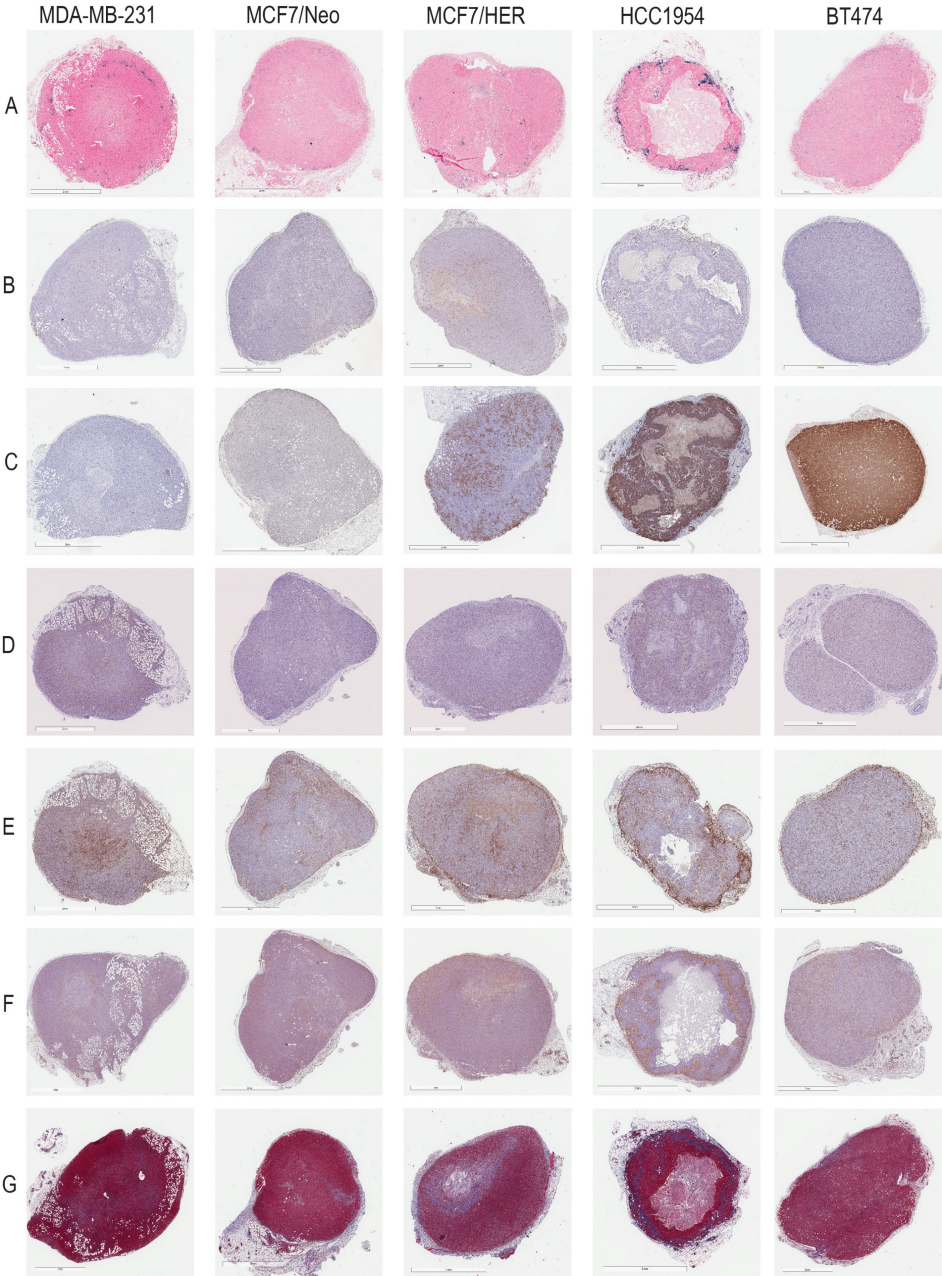
** Representative images for each staining in NSG xenografts.** A. Prussian blue staining for nanoparticles. B. CD31 for blood vessels. C. HER2 for tumor cells. D. CD11c for dendritic cells and other proinflammatory cells. E. F4/80 for macrophages. F. SMA for fibroblasts. G. Masson's Trichrome for collagen.

A detailed schema of the study is given in Figure 1 and S1. A representative image for each staining in xenografts grown in nude and NSG mice are shown in Figure 2 and Figure 3 respectively.

### More antibody-labeled nanoparticles were retained in tumors

BH nanoparticles accumulated significantly higher in all tumors than was observed with BP treatment 26. Amounts of BP (plain or unlabeled) nanoparticles, visualized from Prussian blue stained slides, were negligible. Hence, detailed analysis could proceed only with BH stained slides.

### BH nanoparticle retention was concentrated at tumor periphery

**Figure 4:**
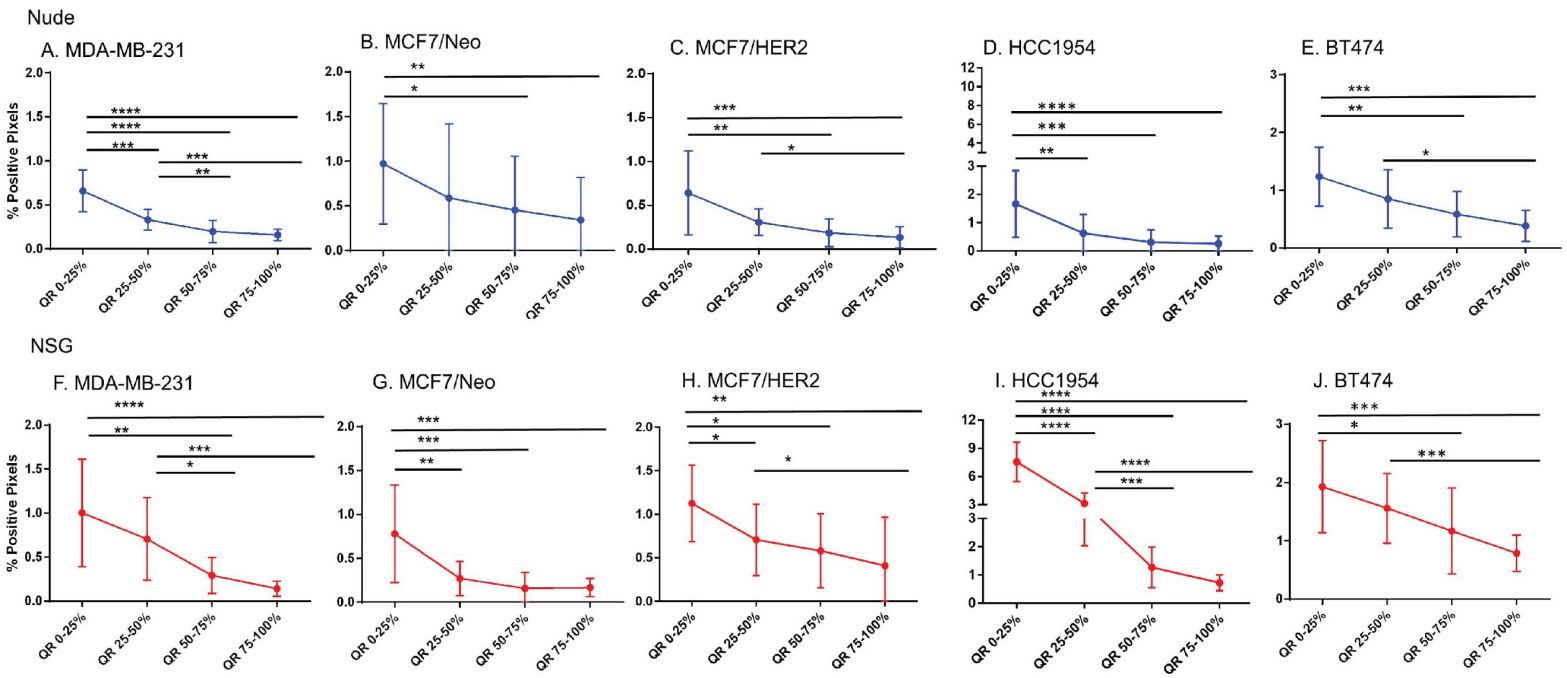
** Significantly different spatial distribution of nanoparticles within tumor.** A -E. All xenografts grown in nude mice had significant differences in nanoparticle accumulation in the tumor when compared from periphery to center. More nanoparticles were accumulated in outer quartiles than inner quartiles. Only a few positive pixels were detected in the inner most quartile. F-J. Similar pattern of distribution was observed in tumor grown in NSG mice as well. (Mann Whitney - *p<0.05; **p<0.01; ***p<0.001).

Equal area quartiles show distinct nanoparticle distribution patterns within each tumor. Irrespective of tumor model, a significantly higher amount of antibody-conjugated nanoparticle was retained in the tumor periphery, decreasing towards the center in subsequent quartiles (Figure 4). Differences among the tumor models grown in the two strains of mice, however were observed and these are described below.

*Xenografts in nude mice:* For MDA-MB231 tumors grown in nude mice (Figure 4A), the outermost quartile retained a significantly higher amount of nanoparticles than did the remaining three quartiles. Additionally, the second quartile had moderately higher amounts of nanoparticles than did the third or fourth inner quartiles. Similarly, MCF7/Neo xenografts had significantly higher nanoparticle retention on the outermost quartile than did the inner third and fourth quartiles (Figure 4B). No significant differences were observed among other quartiles for MCF7/Neo tumors. MCF7/HER2 tumors (Figure 4C) showed a similar pattern of nanoparticle retention; however, the second quartile also had significantly higher nanoparticle accumulation than did the inner third and fourth quartiles. Likewise, HCC1954 xenografts (Figure 4D) showed a similar pattern of nanoparticle accumulation, with higher accumulation in the periphery than any of the inner quartiles. BT474 xenografts (Figure 4E) also displayed significantly higher nanoparticle accumulation in the first and second versus inner quartiles.

*Xenografts grown in NSG mice:* As observed with tumors grown in nude mice, tumors grown in NSG mice also showed significantly higher peripheral accumulation of nanoparticles (Figures 4F-4J). All xenografts showed a significantly different distribution of nanoparticles between the first and inner quartiles. MDA-MB-231, MCF7/HER2, HCC1954 and BT474 had higher pixel positivity in the second quartile than the innermost quartile. An overall increase in the percentage of positive pixels was observed in tumors collected from NSG mice versus those collected from nude mice.

### Host dependent differences in nanoparticle accumulation

**Figure 5:**
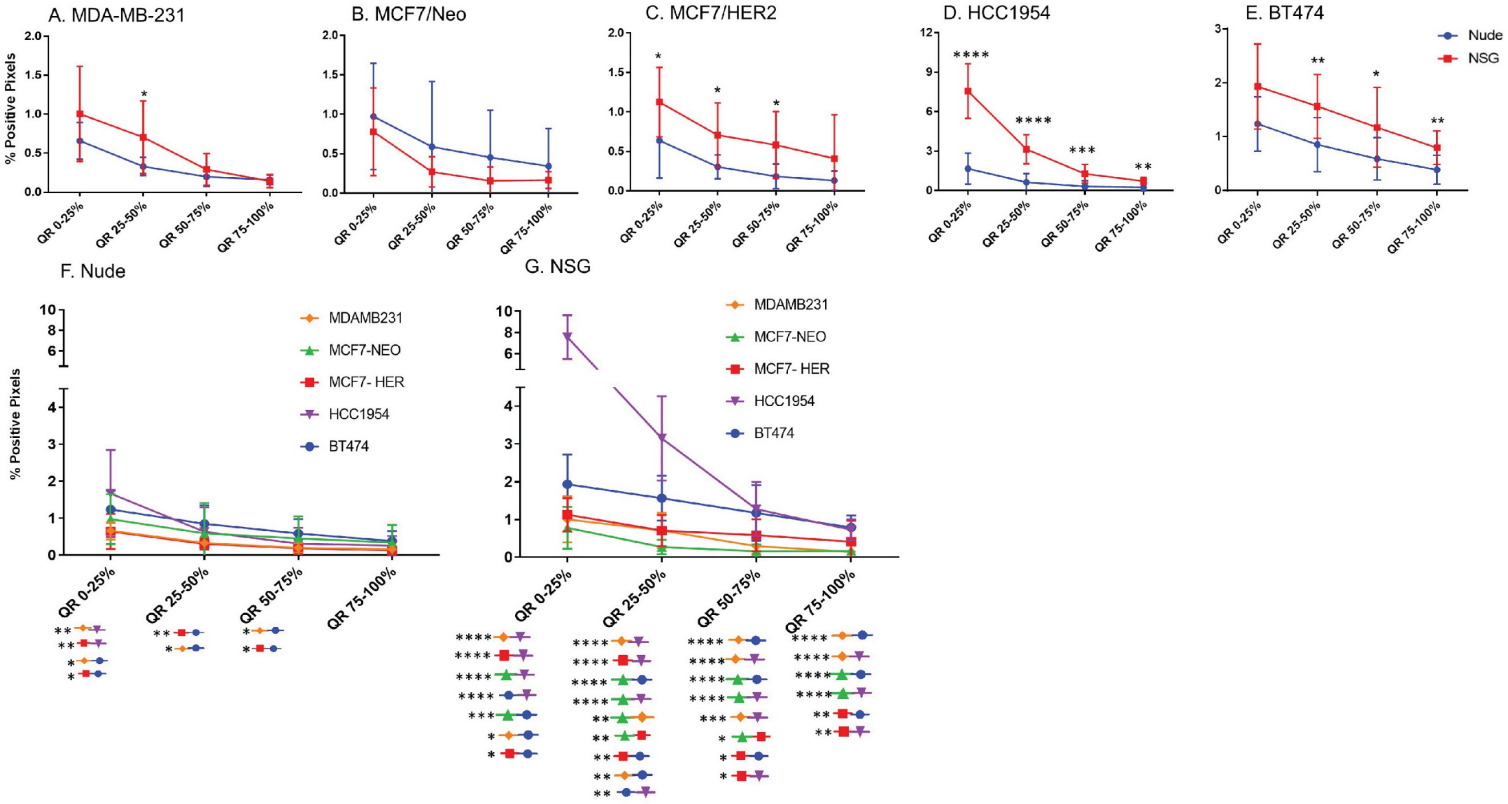
** Significant difference in spatial distribution of nanoparticles in same xenograft grown in nude vs NSG mice.** A -E. HER2 positive xenografts (MCF7/HER2, HCC1954 and BT474) grown in NSG mice had significantly higher nanoparticle uptake in almost all quartiles in tumor when compared from periphery to center whereas HER2 negative xenografts did not show that difference except for one quartile in MDA-MB-231 tumor. F&G. Irrespective of HER2 status, significant differences were observed in different quartiles when compared between xenograft types. This difference was elevated in xenografts grown in NSGs than in nudes. (Mann Whitney - *p<0.05; **p<0.01; ***p<0.001; ****p<0.0001).

Next, we compared differences between nanoparticles in each quartile of the same tumor type when grown in two different mouse strains. As seen in Figure 5, excluding MCF7/Neo, a greater nanoparticle retention was observed in tumors grown in NSG than those grown in nude mice (Figures 5A-E). A significantly higher difference was observed for the three HER2 positive xenografts - MCF7/HER2, HCC1954 and BT474. When comparing among tumors obtained from athymic nude mice, we observed a significantly higher accumulation of nanoparticles on the outer quartile for HCC1954 and BT474 tumors when compared to MDA-MB-231 and MCF7/HER2 tumors (Figure 5F). In the second and third quartiles, there was increased accumulation in BT474 tumors compared to MDA-MB-231 and MCF7/HER2 tumors. No other groups showed differences.

The variation in nanoparticle accumulation among quartiles was more pronounced in tumors grown in NSG mice (Figure 5G). All groups displayed significantly lower nanoparticle retention than HCC1954 on the outer peripheral quartile. Additionally, all groups except HCC1954 had lower nanoparticle retention than did BT474 tumors on the outer quartile. Likewise, in the second quartile, both HCC1954 and BT474 had significantly higher nanoparticle accumulation/density than did any other group. MCF7/Neo had significantly lower nanoparticle density in the second quartile than did either MDA-MB-231 or MCF7/HER2. In the third quartile, both HCC1954 and BT474 had higher nanoparticle accumulation than did the corresponding quartiles in the other tumor. MCF7/HER2 had significantly higher nanoparticle content than did MCF7/Neo in the third quartile. In the innermost quartile, only HCC1954 and BT474 had significantly higher nanoparticle accumulation than did any of the other groups.

### Spatial distribution of stromal cells was similar to nanoparticles

To test the hypothesis that nanoparticle accumulation correlated with spatial distributions of tumor cells or other cell types in the stroma, we performed a similar analysis using IHC stained slides for each cell type.

**Figure 6:**
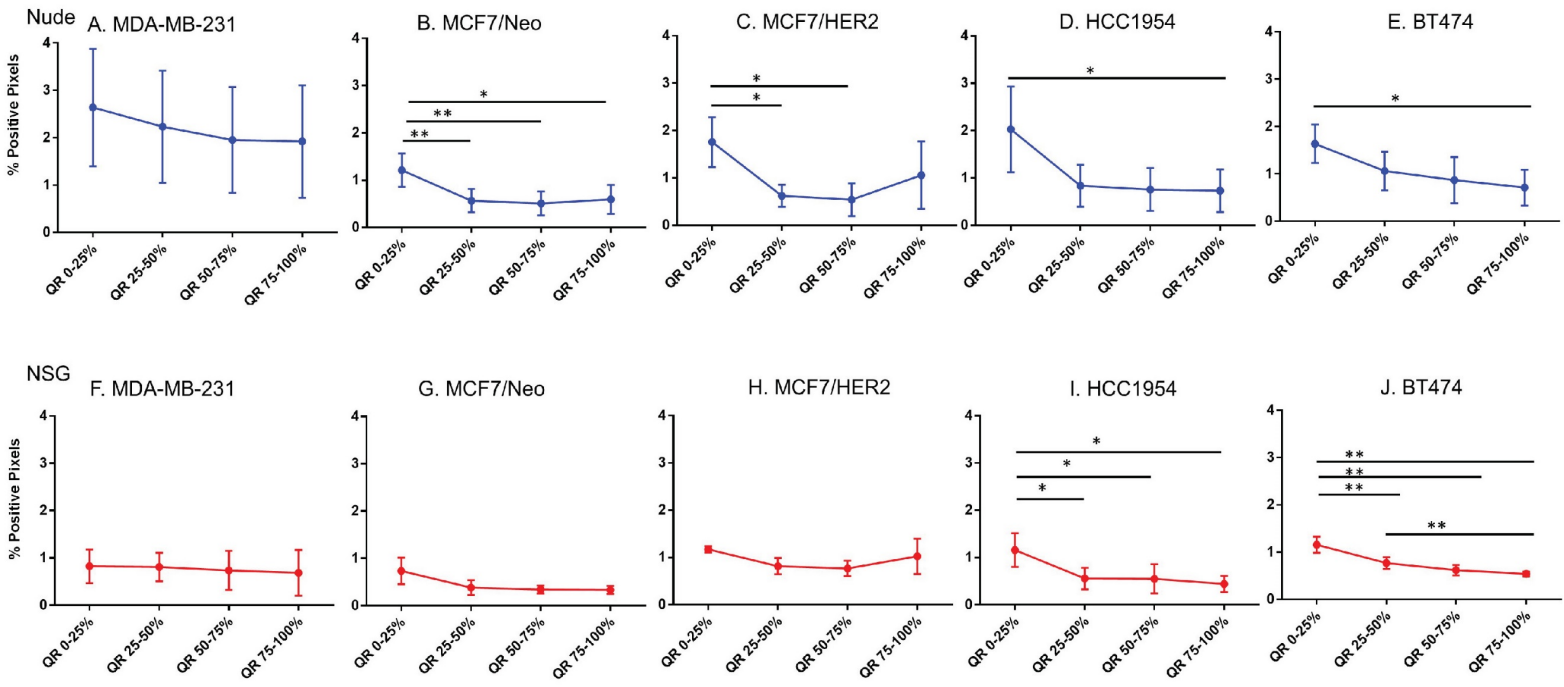
** Spatial distribution of endothelial marker for blood vessel - CD31 in xenografts.** A -E. A significant difference in CD31 staining was found between the outer and inner-most quartiles in most xenografts. MDA-MB-231 did not show any difference in CD31 positive staining. Likewise, MDA-MB231, MCF/Neo and MCF7/HER2 (F-H) grown in NSG did not show any significant difference in CD31 staining between any quartiles. Both HCC1954 and BT474 had a pattern of higher staining on outer quartiles than inner ones. (Mann Whitney - *p<0.05; **p<0.01).

**Figure 7:**
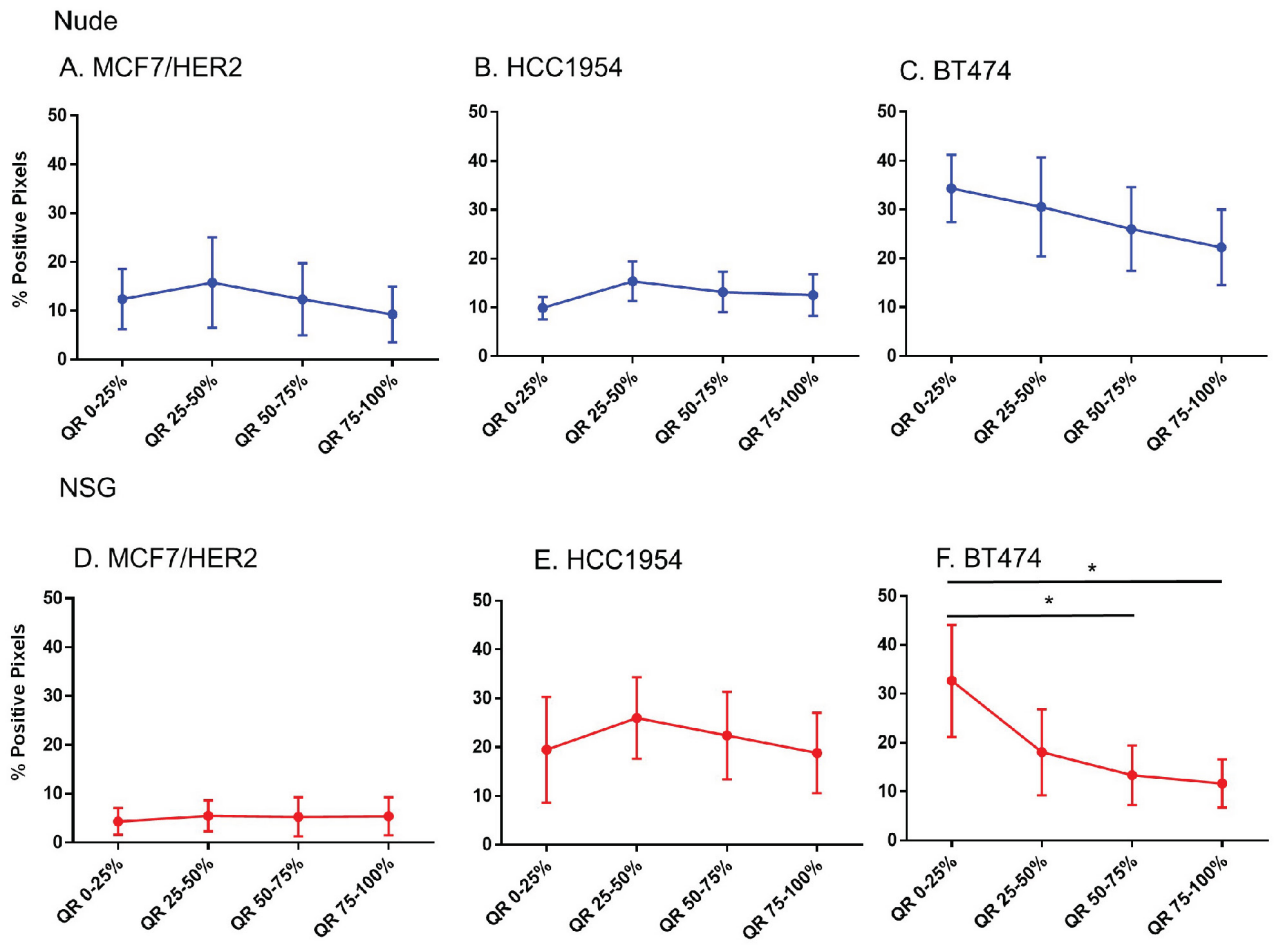
** Spatial distribution of HER2 +ve tumor cells by HER2 staining in xenografts.** Except for BT474 in NSG, HER2 staining was uniform throughout tumors in both Nude and NSG mice growing HER2 overexpressing tumors. (Mann Whitney - *p<0.05).

**Figure 8:**
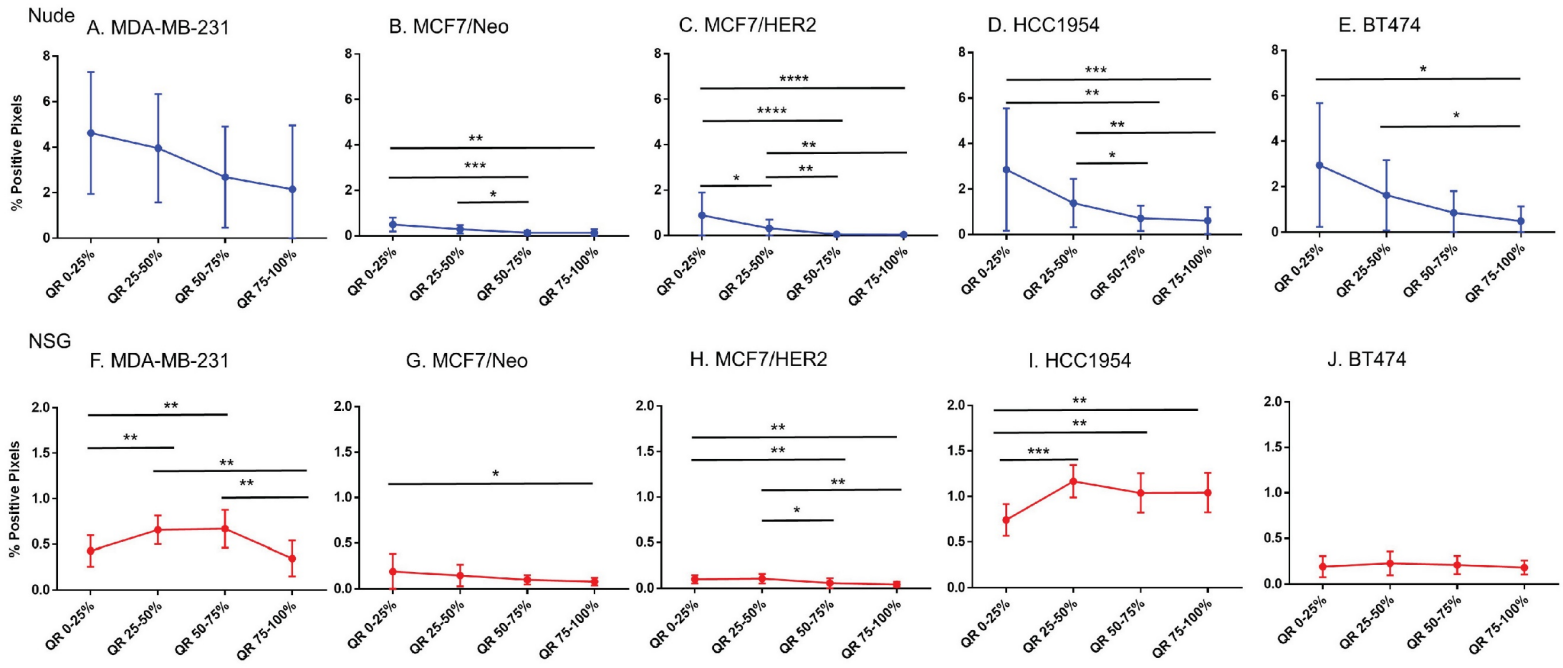
** Spatial distribution of proinflammatory and dendritic cells by CD11c staining in xenografts.** CD11c showed variable distribution from edge towards inner side in almost all tumor types except for MDA-MB-231 grown in nude and BT474 grown in NSG mice (Mann Whitney - *p<0.05; **p<0.01; ***p<0.001; ****p<0.0001).

**Figure 9:**
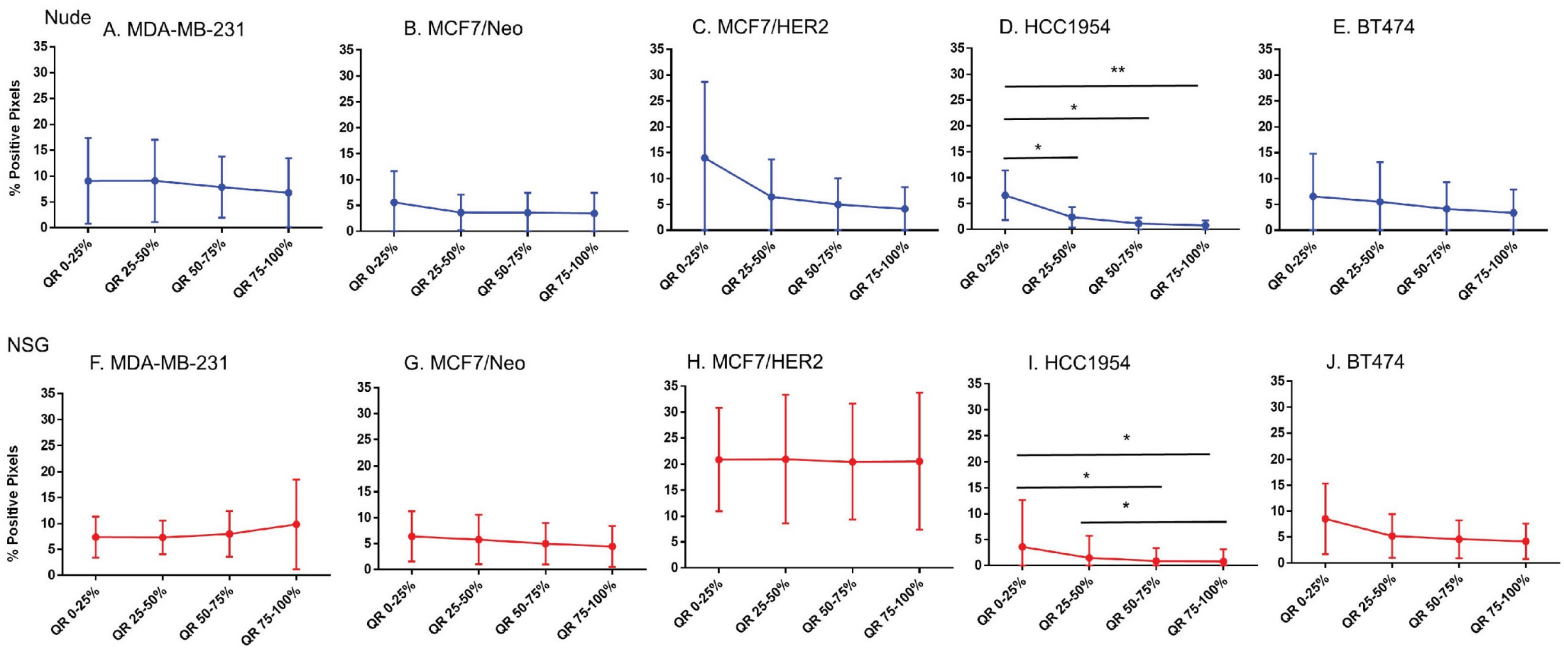
** Spatial distribution of macrophages stained by F4/80 in xenografts.** Macrophage distribution was significantly different within the tumor only in HCC1954 tumors in both nude and NSG mice from the edge towards the center. (Mann Whitney - *p<0.05; **p<0.01).

**Figure 10:**
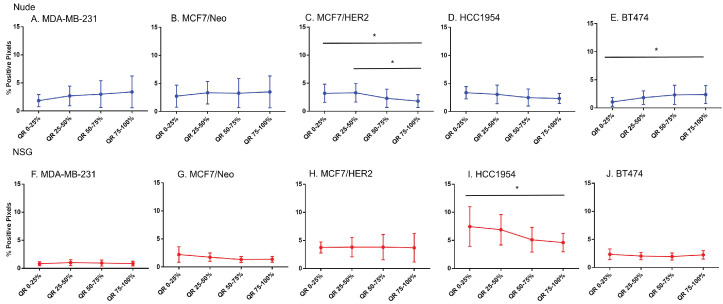
** Spatial distribution of fibroblasts by SMA staining in xenografts.** Minimal distribution changes was observed with α-SMA staining in MCF7/HER2 tumors and BT474 tumors in grown nude mice and HCC 1954 tumors grown in NSG mice (Mann Whitney - *p<0.05).

**Figure 11:**
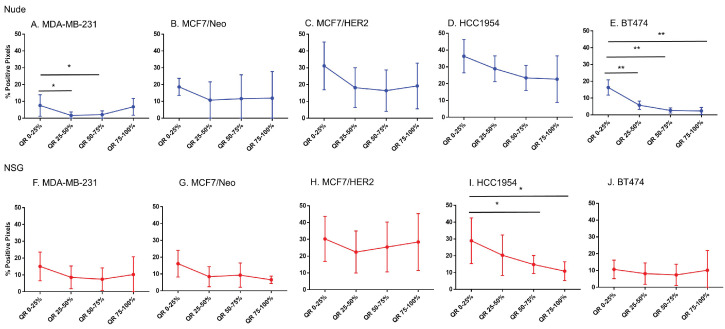
** Spatial distribution of collagen by Masson's trichrome staining in xenografts.** Masson's trichrome showed significant difference in distribution in MDAMB231 and BT474 tumors grown in nude and HCC 1954 grown in NSG mice. (Mann Whitney - *p<0.05; **p<0.01).

As shown in Figures 6-11, overall, there were significant differences of cell distribution within the tumor, depending on both the tumor model and mouse strain.

### Blood vessel density does correlate poorly with nanoparticle distribution in all tumor types

For intravenously injected nanoparticles, the primary route of entry into the tumor is through blood vessels. A staining density analysis of CD31+ regions is a marker of vascular density in the tumor microenvironment. Substantial differences in this endothelial marker indicates variable blood vessel density among tumors and can be used to infer this relationship with uptake of nanoparticles to test hypotheses. As shown in Figure 6, MDA-MB-231 tumors grown in both nude and NSG mice showed little difference in CD31 staining among quartiles even though nanoparticle distribution was significantly different. On the other hand, other tumor MCF/Neo, MCF7/HER2, HCC1954 and BT474 tumors grown in nude mice, and HCC1954 and BT474 grown in NSG mice showed significant difference in positivity from periphery to the center, mirroring that of Prussian blue stained pattern(s) of quartile positivity (Figure 6).

### No difference in CD31 positivity was observed between tumors grown in nude and NSG mice

We then sought to determine if a measurable difference in the distribution of CD31 positive cells could explain patterns of nanoparticle retention observed in tumors grown in the two mouse strains. No significant differences were observed in CD31 positive cells of tumors grown in nude or NSG in any quartile, except for BT474 on the outer quartile (Figures S2A-E). Some differences in CD31 positive cells were observed between MCF7/Neo and BT474 tumors in either nude or NSG as shown in Figures S2F-G. Overall, there was a slight increase in numbers of CD31-positive cells in tumors grown in nude mice than those grown in NSG mice.

These results do not support a hypothesis that increased vascularity contributed to the higher accumulation of nanoparticles found in tumors grown in NSG mice versus those grown in nude mice. Furthermore, if the blood vessel density was a driver of nanoparticle accumulation based on expectations from EPR, we expect this to also have been observed in BP treated mice. As noted, little accumulation of BP nanoparticles in any of the tumor types, measured by both ICPMS and Prussian blue, was observed 26.

### No difference in HER2 positivity among quartiles were observed in HER2 tumors, except BT474 tumors grown in NSG mice

That only BH nanoparticles accumulated in substantial amounts in any tumors, and that all HER2+ tumors showed BH retention, led to a hypothesis that perhaps antibody-antigen binding to cancer cell membranes of HER2+ tumors was a factor. To test this hypothesis, we measured whether Herceptin®-labeled nanoparticle retention correlated with distributions of the target antigen - HER2 expression, in the tumor microenvironment. We analyzed nanoparticle distribution patterns in each quartile corresponding to the target antigen expression.

Except in BT474 tumors grown in NSG mice, no significant differences in the HER2 intensity for each quartile among any of HER2+ve models was observed (Figure 7). Comparing among tumor models grown in nude versus NSG mice, no significant differences in positive staining were observed to explain the higher nanoparticle retention in the latter for HER+ve tumor types (Figure S3). In fact, there seemed to be a higher positivity for tumors in nude mice than NSG for MCF7/HER2 and BT474 tumors that failed to translate to increased nanoparticle retention. Furthermore, nanoparticle distributions observed in sections obtained from HER2-negative tumors were appreciably similar to those obtained from HER2-positive tumors.

### CD11c staining was variable among tumor types and between mouse strains

Considering increased BH (Herceptin®-labeled) nanoparticle distributions correlated poorly with markers of vascular density or target antigen expression, we sought to test additional hypotheses that other cell phenotypes were responsible for the observed nanoparticle accumulation. Phagocytic (innate immune) cells take up nanoparticles, particularly circulating nanoparticles by macrophages circulating in blood and those residing in liver, spleen, intestines, and other tissues. Further, myeloid derived innate immune cells exhibiting a proinflammatory phenotype display enhanced tendency to uptake BH nanoparticles in vitro 26. Thus, we hypothesized that the state of immune activation in either tumor or host can influence retention.

From comparisons of slides stained with CD11c that stains dendritic cells and some proinflammatory cells in the tumor microenvironment, we observed significant differences in immune cell distributions among the quartiles of all tumor types (Figure 8). In addition, differences between same tumors grown in nude and NSG mice were also observed. All tumors grown in nude mice had comparatively higher CD11c positive cell staining than their counterparts grown in NSG mice for each quartile. Significant differences in positivity between tumors grown in nude and NSG mice were also observed (Figure S4). Nude mouse tumors had significantly higher positivity in the outer quartile than those of same tumors grown in NSG.

### Macrophage distribution varied by quartile in HCC 1954 tumors

Analysis of the quartile distribution of macrophages in all tumors revealed that significant differences among quartile distributions was observed only in HCC1954 tumors (Figure 9). Significant differences between nude and NSG mice were observed in MCF7/HER2 and HCC1954 tumors (Figure S5).

### Fibroblast cell and collagen distribution in tumors significantly varied among quartiles

MCF7/HER2 and BT474 tumors grown in nude mice and HCC1954 tumors grown in NSG mice showed significant differences in their distribution of fibroblasts among all quartiles (Figure 10). When we compared the quartile positivity of fibroblast cells between corresponding tumors grown in nude or NSG mice, HCC1954 in NSG mice showed significantly higher positivity in all quartiles than did the same tumors grown in nude mice (Figure S6). The outer quartile of MDA-MB-231 and BT474 also showed significant differences between the two mouse backgrounds.

MDA-MB-231 and BT474 tumors grown in nude mice and HCC1954 in NSG mice showed significant differences in positivity for collagen among their quartiles (Figure 11). Except for MDA-MB-231 and BT474 in one quartile, no significant differences were observed between the same quartile of corresponding tumors grown in the two mouse strains (Figure S7).

### Normalized pixel positivity shows positive correlation between nanoparticle and stromal cell distributions

**Figure 12:**
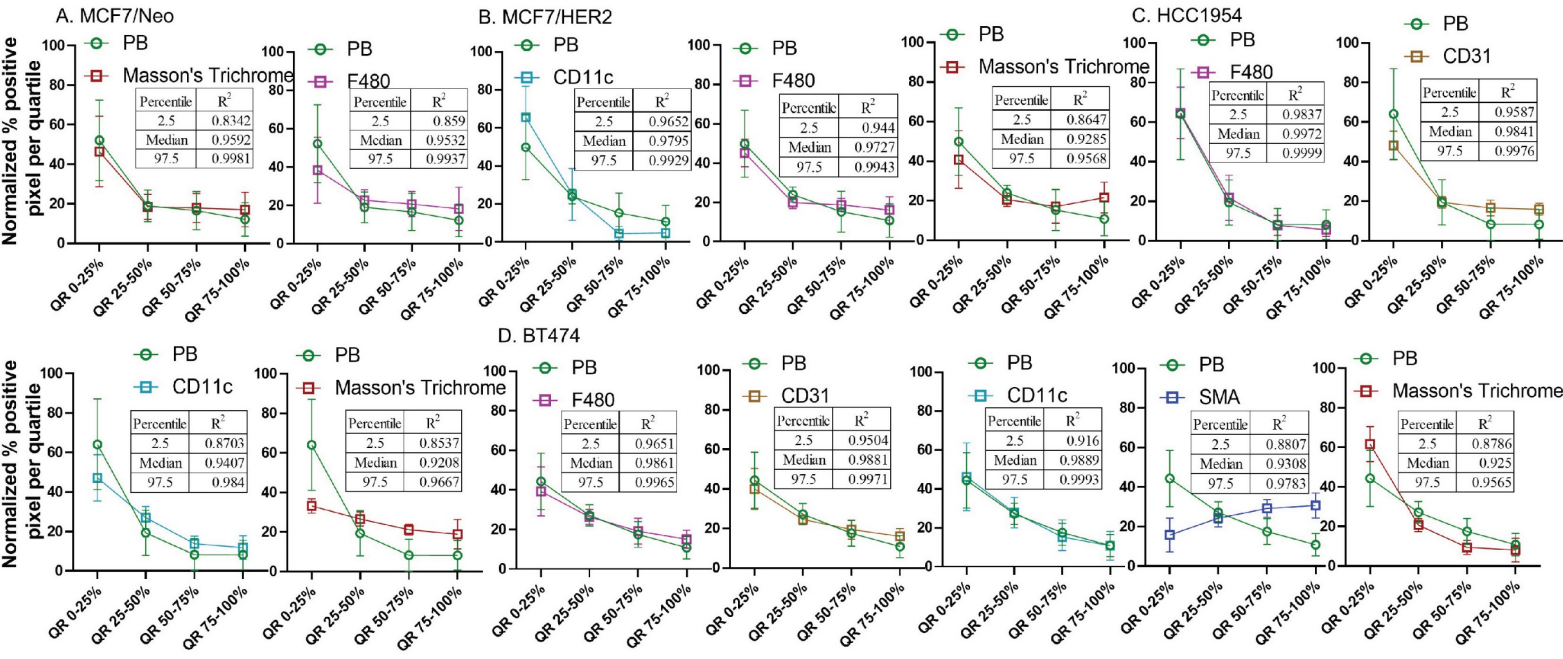
** Spatial distribution of Prussian blue stained nanoparticle areas in tumor correlated with immune or stromal cells in xenografts grown in nude mice.** Positive pixels within each quartile was normalized with respect to the total amount of positive pixels with each stain present within tumors grown in nude mice. Consistently higher percentage of nanoparticle and cell population were found in the outer quartile of nude xenografts. Prussian blue (PB) percentages among the four quartile generally decrease from the outer quartile to the inner 50% of the tumor. Varying pattern of distribution was seen for each cell type specific stains. MDA-MB 231 tumor PB does not correlated strongly with any of the cell types. A. In MCF7/neo tumors, the correlation was high with collagen stained by Masson's trichrome and macrophages stained for their marker F4/80. B. In HER2 overexpressing MCF7 tumors, MCF7/HER2, nanoparticle distribution correlated most with proinflammatory and dendritic cell (CD11c) distribution, macrophages (F4/80) and collagen (Masson's Trichrome). C. In another HER2+ve tumor, HCC1954, nanoparticle distribution correlated with that of macrophage distribution (F4/80), blood vessel density (CD31), CD11c (dendritic and proinflammatory cells) and collagen (Masson's Trichrome). D. Interestingly, in highly HER2 overexpressing BT474 tumors nanoparticle distribution does not correlated with HER2 but had high correlation with that of macrophages (F4/80), blood vessels stained by endothelial marker CD31, proinflammatory and dendritic cells (CD11c), fibroblast marker SMA and collagen (Masson's Trichrome).

From the observed results, we hypothesized that certain stromal cell types, depending on the tumor model and mouse background, were key players in determining nanoparticle retention in the tumor microenvironment. To better standardize and compare the gathered data, we normalized the pixel positivity of each quartile to the total positive pixel count in each tumor for all stains. Table 1-5 shows the 2.5 percentile, 50 percentile, and 97.5 percentile of the 1000 bootstrap sample R^2^ calculated. Depending on the R^2^ value, the best-correlated cell type with that of nanoparticle distribution of each xenograft is shown in Figure 12. MDA-MB-231 xenografts in nude mice showed a weak correlation with any of the cell types analyzed here. In MCF7/neo tumors, correlation between nanoparticles and collagen or macrophages were strong. HER2 positive MCF7/HER2 xenografts showed nanoparticle distribution highly correlated with dendritic cells, macrophages and collagen distribution. Nanoparticle distribution in another HER2 overexpressing HCC1954 tumors also correlated strongly with that of macrophages, blood endothelial cells, dendritic/proinflammatory cells and collagen. Likewise, HER2 amplified BT474 xenografts also showed strong correlation between nanoparticles and macrophages, endothelial cells, dendritic/proinflammatory cells, fibroblasts and collagen. It is noteworthy that none of the HER2 positive xenografts showed a strong correlation between targeted nanoparticles and the target antigen - HER2.

**Figure 13:**
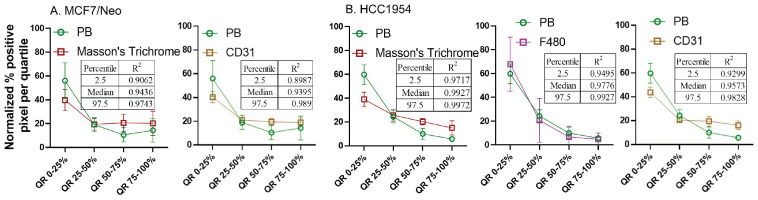
** Spatial distribution of Prussian blue stained nanoparticle areas in tumor correlated with stromal cells in few tumor types grown in NSG mice.** Nanoparticle distribution does not correlate with any cell types in MBA-MB-231, MCF7/HER2 or BT474 xenografts grown in NSG mice A. Nanoparticle distribution (PB) in MCF7/neo tumors correlated with collagen (Masson's trichrome) and blood vessels (CD31). B. In HER2+ve HCC1954 tumors, nanoparticle distribution correlated most with that of collagen distribution (Masson's Trichrome), macrophages (F4/80) and blood vessels (CD31). As observed in HER2+ve nude xenografts, no correlation was found with HER2 distribution and PB in any of the xenografts.

Overall, four out of five tumor types showed a higher correlation with macrophage and collagen distribution in tumors collected from nude mice. When we evaluated tumors grown in NSG mice, collagen content and endothelial cells correlated best with nanoparticle distribution in the corresponding slides from MCF7/neo and HCC1954 xenografts (Figure 13). In addition, HCC1954 tumors showed a high positive correlation of nanoparticle distribution with macrophages as well. MDA-MB-231, MCF7/HER2 or BT474 xenografts showed weak correlations with any of the cell type analyzed in NSG mice (Figure S8-S12). Here too no correlation was seen between HER2 positive tumor cells and nanoparticle distribution despite having higher accumulation of BH particles in HER2+ve NSG xenografts.

## Discussion and Conclusion

Despite advances in understanding the properties of nanostructured materials, achieving consistent, targeted delivery of an engineered nanoparticle to solid tumors for theranostic applications remains unaccomplished. Current designs of nanoparticle agents rely heavily on tuning the physicochemical properties of the material to a passive diffusion-dominated mechanism, rather than the biological characteristics of the host or disease. Both the biology of the host and disease play a crucial role in the fate of systemically administered nanoparticles. We can expect that once in the bloodstream, nanoparticles interact with blood proteins. Thus, their uptake and accumulation in various organs we can hypothesize is dictated, in part, by individual cell interactions with the protein corona 27, 28.

Regardless of surface modification, the liver and spleen sequester most nanoparticles as part of their function 29. To isolate and entrap foreign bodies, these organs are enriched with phagocytic cells that engulf foreign materials. Increasing circulation time in the bloodstream by “stealth” coating can modestly improve accumulation of some nanoparticle formulations in some tumor models, but this relationship lacks consistency among tumor models as barriers in the tumor microenvironment that are defined by biology, actively prevent interactions with target tumor cells. Furthermore, individual variations seem to preclude a broader translation of these observations into clinical reality 30, 31.

To understand better the challenges posed by host and tumor biology, we considered that a further analysis of interactions between nanoparticles and host cells/tissue at a microscopic level was needed. Here, we evaluated the interaction between a plain (no antibody) and an antibody-coated nanoparticle and the tumor microenvironment among several models (representing antigen positive and negative tumor biology) across two different immune strains of mouse by conducting a comparative spatial analysis of tissue histology sections. We measured no appreciable retention of unlabeled nanoparticles in the tumor microenvironment providing little evidence supporting a passive diffusion-dominated retention mechanism. In an earlier study, we documented that the antigen-antibody interaction mediated uptake of antibody-conjugated nanoparticles to the tumor cells, while is significant *in vitro*, is negligible *in vivo* 26. Conversely, for the antibody-labeled nanoparticles, we found that irrespective of tumor biology, the highest accumulation of nanoparticles occurred at the periphery of the tumor and decreased towards its center. This was equally consistent across tumor models that lacked or expressed the antigen, even if antigen expression was constant throughout the tumor.

This suggested to us that a complex interplay of biological and physical factors exist to influence uptake and retention of nanoparticles in the complex environment within tumors. We also report that among HER2-overexpressing tumor models (MCF7/HER, HCC1954 and BT474) grown in two different immune strains of mouse, significantly different patterns of nanoparticle retention were observed, despite the similarities of blood vessel density measured among them. These data directly challenge the hypothesis that vascularity and access to target-expressing cancer cells is a primary factor that determines targeted-nanoparticle retention in a tumor. In other words, nanoparticles circulating in blood gain access to tumors as well as to all other tissues, but whether they remain in a specific (target) tissue depends on host or other potentially independent biological factors comprising the local tissue environment. Evaluation of different cell types in the tumor microenvironment revealed that in each tumor type, whether grown in nude or NSG mice, dominant cell types residing in the tumor dominated retention. Further analysis illustrated that in tumors grown in nude mice, it was the distribution of resident macrophages and collagen content that correlated predominantly with nanoparticle distributions within each tumor quartile. On the other hand, very few correlations between nanoparticle distribution and cell type were identified in tumors grown in NSG mice. Little correlation between nanoparticle localization and HER2 positive regions were documented in both strains and among three different human HER2-positive tumor grafts displaying varying amounts of HER2 expression. These findings highlight the need for understanding the complexity of the biological system for targeted delivery of nanoparticles. We visualized and analyzed collagen with Masson's trichrome staining. This is the most common method used in both research and clinical settings to visualize tissue collagen content and distribution, but it provides little information on the structural differences and types of collagen fibers in each tumor type in different strains of mice. Such structural differences in collagen fibers may influence nanoparticle retention in tumor microenvironment, and may merit further investigation. To analyze those differences, other high resolution and/or phase contrast imaging modalities such as those employing second harmonic imaging, coherent anti-Stokes Raman scattering (CARS) microscopy, etc. may prove beneficial 32-33.

Increased accumulation of nanoparticles in the tumor periphery has been documented with other i.e., liposomal nanoparticle types, which is usually attributed to increased vascular density within the tumor 34. Although this may explain nanoparticle retention in some tumor models; and for certain nanoparticles, here we demonstrated that variability among tumor types failed to yield a positive correlation of blood vessel density with retention of the BH nanoparticles in most xenografts analyzed. Our results also show that a significantly higher percentage of nanoparticles accumulated in MCF7/HER2, HCC1954 and BT474 grown in NSG mice than those grown in nude mice, despite the vascular density being comparatively higher in the latter. This pattern is counter to that predicted by an explanation that vascular access is the dominant or significant factor determining nanoparticle retention in tumors. Evidently, differences among the tumors grown in different hosts indicates additional and potentially complex biological factors operate to determine nanoparticle fate, going well beyond vascular density of tumors.

The phenotype displayed by phagocytic cells when they encounter nanoparticles can significantly alter the quantity of nanoparticle they will ingest. Pro-inflammatory (M1) macrophages or induced neutrophils ingest significantly higher amounts of both antibody-conjugated and unconjugated nanoparticles than their uninduced counterparts 26. Gene expression studies have further revealed that the tumor periphery often displays a more pro-inflammatory phenotype, i.e., a balance of immune cell populations significantly “tilted” towards these phenotypes, than the surrounding normal tissue 35. Hence, we speculate that nanoparticles delivered via blood to the tumor microenvironment are more likely to be retained at the tumor periphery if this region harbors significant populations of pro-inflammatory phagocytic cells. This, in turn can increase nanoparticle accumulation in the tumor periphery compared to the tumor center. Blood vessel size, permeability, and interstitial pressure, as well as active transport by endothelial cells may further influence nanoparticle access to the tumor microenvironment, which demands further investigation 24, 36.

Our analysis suggests that the spatial distributions of many stromal cell types making up the tumor microenvironment correlated better with the observed distribution of trastuzumab-labeled BNF^®^ nanoparticles retained in the tumor, than did that of the HER2-overexpressing tumor cells. Moreover, when the mouse strain changed, the cell type that predominantly correlated with the nanoparticle distribution in the tumor also changed. We note that the models chosen represent human cancer tissue grafts grown on mouse hosts. Thus, tumor stroma, blood, and all other tissues are murine, whereas the cancer cells (HER2 positive or negative) within the tumor are human. While the biological complexities of these models for studies of cancer nanomedicine cannot be underestimated, the benefits to compare among a specific number of variables in living systems can provide important insights. Despite the obvious limitations, use of human tissue xenografts grown in permissive animal hosts, e.g. immune compromised mice provide an important, albeit limited, tool to ascertain potential effectiveness of research pharmaceuticals against human diseases. From another perspective, syngeneic models (i.e., species-specific tissue grafts grown on the immune competent species-matched hosts) yield important information regarding interactions of the pharmaceutical agent with intact, but disease-affected host immune system in the context of the disease model. In such cases, the effectiveness of the agent to treat human disease may not translate directly, but results obtained from such a model (e.g. murine tumor grown in matched strain of mouse) might give us more insight that is relevant to host immune- and stroma-specific influences on agent pharmacodynamics. Further, results obtained from such models require additional caution for interpretation when the studies involve clinically relevant human-specific “immunogenic” agents, such as a monoclonal antibody as used in this study. We anticipate further insights with our ongoing efforts with analysis of mouse tissues following treatment with Herceptin^®^-conjugated iron oxide nanoparticles using tumor grafts obtained from human HER2 overexpressing genetically modified mice.

Another limitation of the study is the limited number of stromal cell lineages we considered at only one time point, and only over a small portion of the tumor. In this study, sizes of tumors were ~ 150 mm^3^ and the influence of tumor volume on outcomes is undetermined. As reported by Sykes et al., 2015, varied tumor pathophysiology with tumor size can influence nanoparticle uptake 37. In addition, we have not considered the absolute amount of nanoparticle that each cell type can take up based on host background and tumor microenvironment.

Despite the limitations, we conclude that host immune-biology, disease, and non-cancer cell composition comprising the tumor microenvironment exert powerful influences to dictate patterns of nanoparticle retention and distribution within solid tumors. A simple, passive and diffusion-dominated mechanism of nanoparticle retention in solid tumors though supported by data, inadequately explains complexities observed. This knowledge is vital for future development of cancer nanomedicine, and may motivate new approaches to query cancer biology with nanotechnology. Additional in-depth computational analyses of the interplay between nanoparticle distribution, host biology, and immune status will enhance our understanding of biomolecular patterns that drive the behavior of nanoparticles within a specific tumor microenvironment. The ability to characterize and predict nanoparticle localization is a crucial first step towards development of theranostic materials.

## Supplementary Material

Supplementary figures.

## Figures and Tables

**Table 1:** R-squared values for MDA-MB-231 xenografts

	Marker	2.5percentile	Median	97.5percentile
**Nude**				
	CD31	0.5352	0.8639	0.999
	Masson's trichrome	0.0763	0.2143	0.3493
	Smooth muscle actin	0.5803	0.9248	0.9855
	F4/80	0.2922	0.4885	0.8153
	CD11c	0.5451	0.72	0.8295
**NSG**				
	CD31	0.2146	0.5828	0.9887
	Masson's trichrome	0.8148	0.8993	0.9397
	Smooth muscle actin	0.0011	0.0481	0.4596
	F4/80	0.4191	0.8027	0.9674
	CD11c	0.0022	0.0231	0.0558

**Table 2:** R-squared values for MCF7/neo xenografts

	Marker	2.5percentile	Median	97.5percentile
**Nude**				
	CD31	0.7405	0.8963	0.9849
	Masson's trichrome	0.8342	0.9592	0.9981
	Smooth muscle actin	0.6669	0.9388	0.9951
	F4/80	0.859	0.9532	0.9937
	CD11c	0.7853	0.8545	0.908
**NSG**				
	CD31	0.8987	0.9395	0.989
	Masson's trichrome	0.9062	0.9436	0.9743
	Smooth muscle actin	0.7207	0.9446	0.9964
	F4/80	0.7236	0.9086	0.9915
	CD11c	0.6614	0.78	0.8472

**Table 3:** R-squared values for MCF7/HER2 xenografts

	Marker	2.5percentile	Median	97.5percentile
**Nude**				
	CD31	0.3803	0.461	0.6528
	Masson's trichrome	0.8647	0.9285	0.9568
	Smooth muscle actin	0.3987	0.5956	0.7373
	F4/80	0.944	0.9727	0.9943
	CD11c	0.9652	0.9795	0.9929
	HER2	0.0318	0.0536	0.1514
**NSG**				
	CD31	0.1777	0.2982	0.3575
	Masson's trichrome	0.1476	0.3739	0.4982
	Smooth muscle actin	0.0335	0.7178	0.9964
	F4/80	0.6912	0.9129	0.9992
	CD11c	0.5021	0.6481	0.8401
	HER2	0.6924	0.7875	0.855

**Table 4:** R-squared values for HCC1954 xenografts

	Marker	2.5percentile	Median	97.5percentile
**Nude**				
	CD31	0.9587	0.9841	0.9976
	Masson's trichrome	0.8537	0.9208	0.9667
	Smooth muscle actin	0.5603	0.7807	0.9235
	F4/80	0.9837	0.9972	0.9999
	CD11c	0.8703	0.9407	0.984
	HER2	0.1586	0.3284	0.5859
**NSG**				
	CD31	0.9299	0.9573	0.9828
	Masson's trichrome	0.9717	0.9927	0.9972
	Smooth muscle actin	0.664	0.6895	0.7936
	F4/80	0.9495	0.9776	0.9927
	CD11c	0.4428	0.5103	0.6856
	HER2	0.0041	0.0224	0.1873

**Table 5:** R-squared values for BT474 xenografts

	Marker	2.5percentile	Median	97.5percentile
**Nude**				
	CD31	0.9504	0.9881	0.9971
	Masson's trichrome	0.8786	0.925	0.9565
	Smooth muscle actin	0.8807	0.9308	0.9783
	F4/80	0.9651	0.9861	0.9965
	CD11c	0.916	0.9889	0.9993
	HER2	0.8213	0.8742	0.9438
**NSG**				
	CD31	0.7286	0.8151	0.8494
	Masson's trichrome	0.5899	0.8963	0.9489
	Smooth muscle actin	0.0135	0.2721	0.8417
	F4/80	0.6344	0.7436	0.8081
	CD11c	0.0096	0.1306	0.4136
	HER2	0.7035	0.8056	0.8372

## Abbreviations

BNF: bionized nano ferrite nanoparticles
BP: bionized nanoferrite plain nanoparticles
BH: bionized nanoferrite nanoparticles conjugated with HER2 antibody
HER2: human epidermal growth factor receptor-2
NSG: NOD SCID gamma
NOD SCID: non-Obese diabetic severe combined immunodeficient
EPR: enhanced permeability and retention
CD: cluster of differentiation
SMA: smooth muscle actin
IACUC: institutional animal care and use committee
DAB: 3,3'diaminobenzidine
IHC: immunohistochemistry
HRP: horse raddish peroxidase
IgG: immunoglobulin
ROI: region of interest
