# Supplementary material for: Spatial analysis of nanoparticle distribution in human breast xenografts reveals nanoparticles targeted to cancer cells localized with tumor-associated stromal cells
Source: Nanotheranostics. 2023 Jun 26;7(4):393–411. doi: 10.7150/ntno.84255 (PMC10327423; doi:10.7150/ntno.84255)
Supplement: Supplementary file 1 — Supplementary figures. [file ntnov07p0393s1.pdf]

Supplementary figures

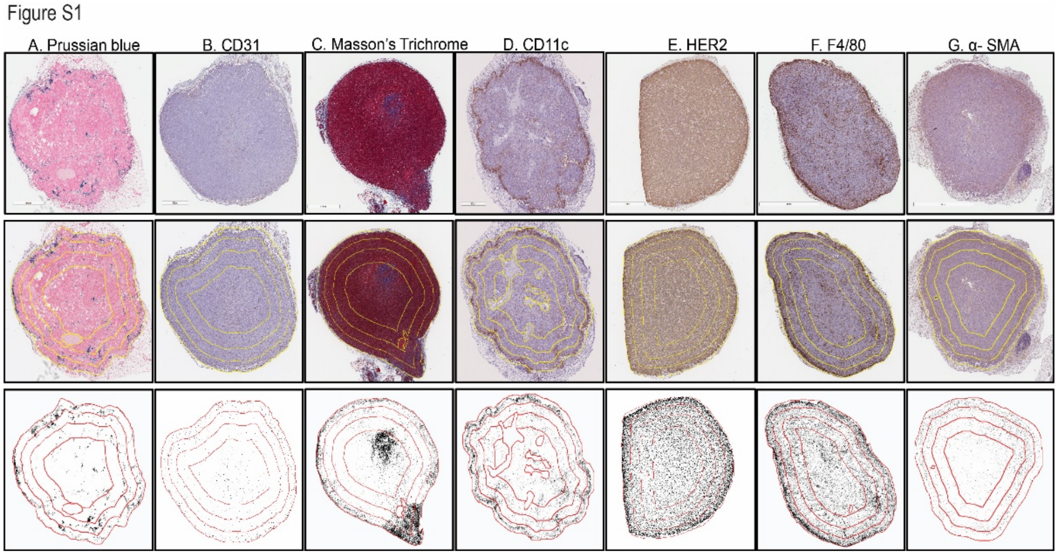

Figure S1: Representative image for each staining in a tumor and their subsequent annotation for region of interest (ROI) and binary conversion for quantification. Necrotic and ambiguous areas were excluded from analysis using negative selection tool in ImageJ/FIJI.

Figure S2

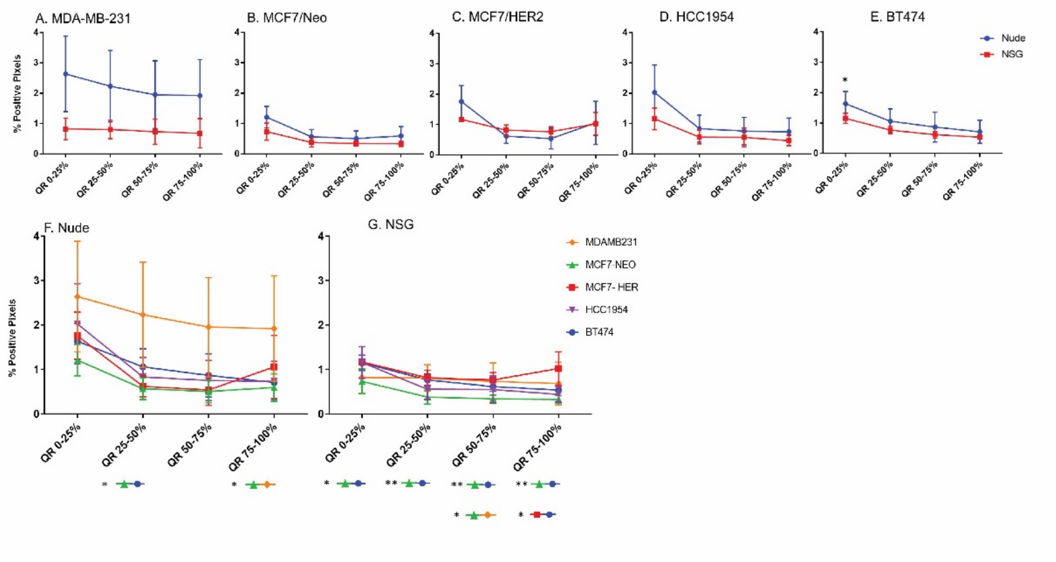

Figure S2: No significant difference in spatial distribution of CD31 positive cells in same xenograft grown in nude vs NSG. A –E. None of the xenografts except BT474 on outer quartile showed any significant difference in CD31 positive staining, which indicates the blood vessel distribution is similar in all xenografts grown in either nude or NSG. F&G. Minimal differences in CD31 positivity were observed in different quartiles when compared between different xenografts grown in nude or NSG. (Mann Whitney - *p<0.05; **p<0.01).

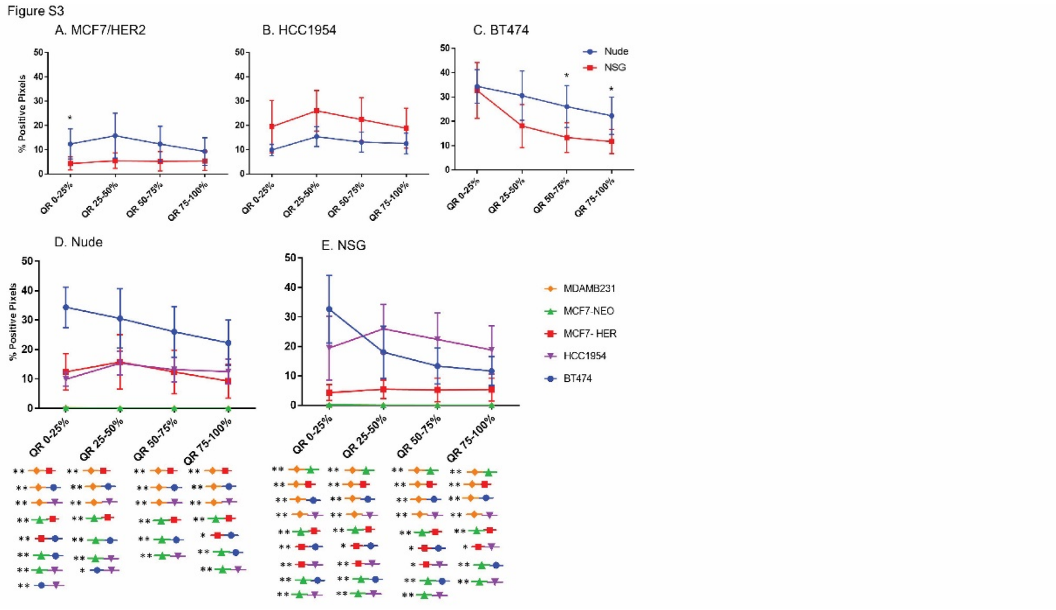

Figure S3: As expected significant difference in HER2 staining was detected in different tumor types with BT474 expressing highest amount followed by HCC 1954 and MCF7/HER2 with background level staining in MDA-MB-231 and MCF7/neo tumors. (Mann Whitney - *p<0.05; **p<0.01).

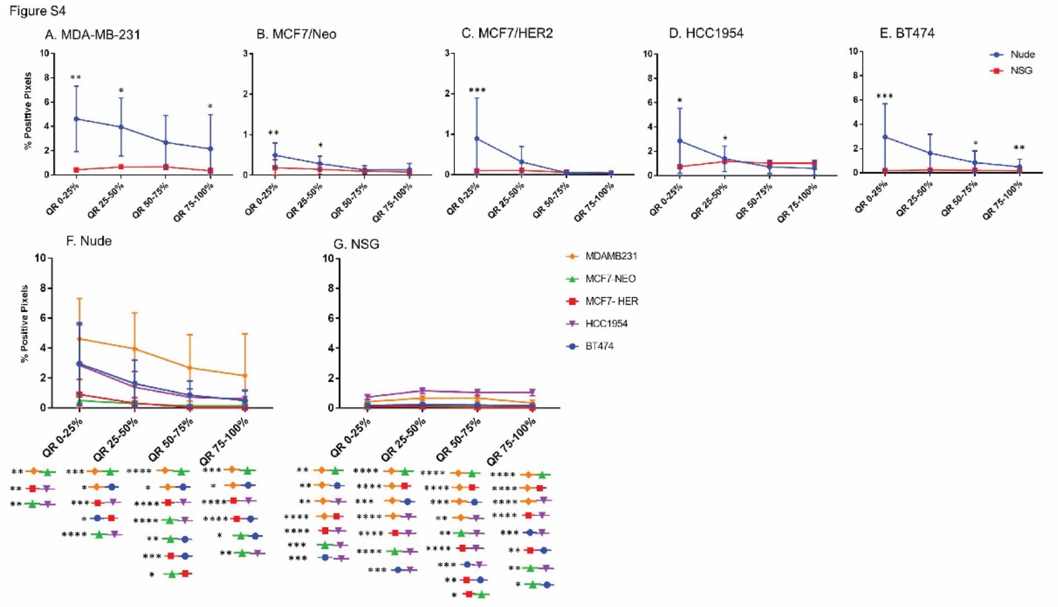

Figure S4: Significant difference in CD11c content was quantified in each quartile when the same tumors were grown in nude vs NSG. Nude mice tumors had higher amount of CD11c staining compared to that of NSG. Significant differences were also detected in each quartile between each tumor types grown in nude and NSG mice. (Mann Whitney - *p<0.05; **p<0.01; ***p<0.001; ****p<0.0001).

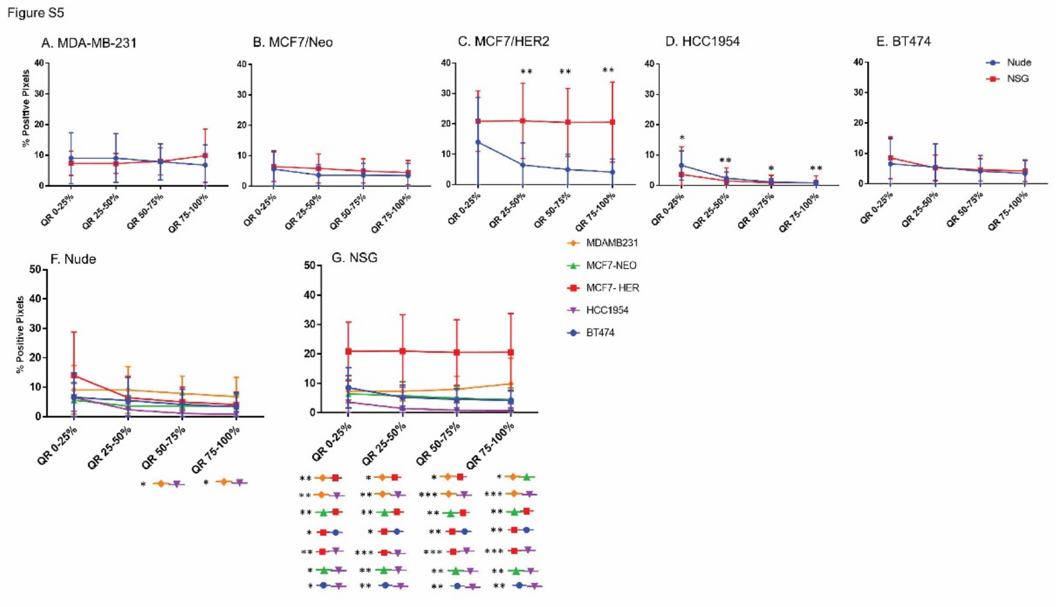

Figure S5: Macrophage content was significantly higher in MCF7/Her2 tumors grown in NSG than nude and vice versa in HCC 1954 tumors. Significant differences in content was observed between tumor types when they were grown in NSG mice (Mann Whitney - *p<0.05; **p<0.01; ***p<0.001).

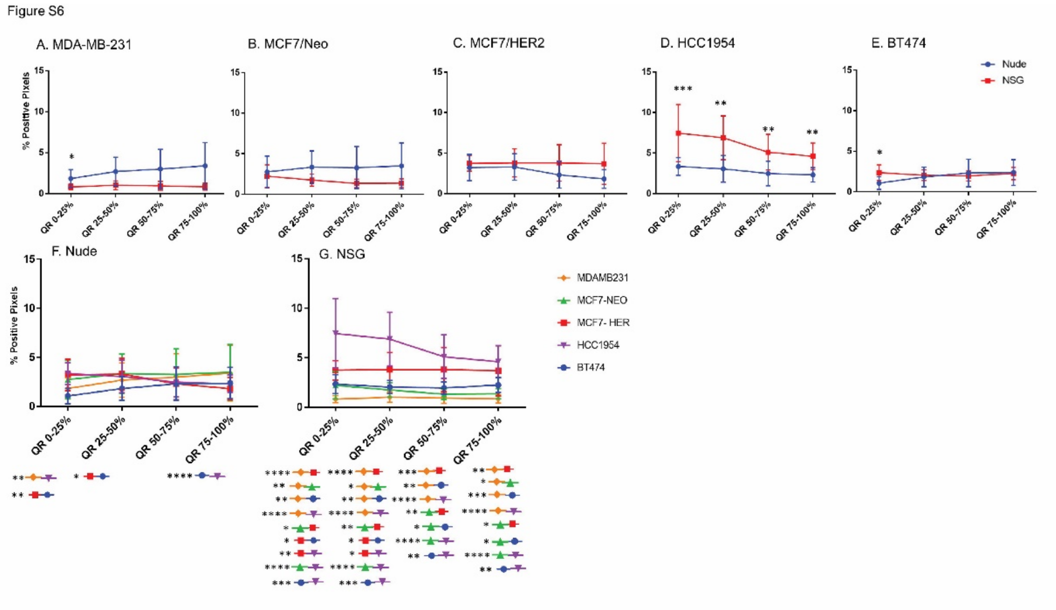

Figure S6: Significant difference in fibroblast content was quantified in HCC 1954 tumors grown in NSG vs nude. Tumors grown in NSG mice had significant variability in SMA content as evident from G (Mann Whitney - *p<0.05; **p<0.01; ***p<0.001; ****p<0.0001).

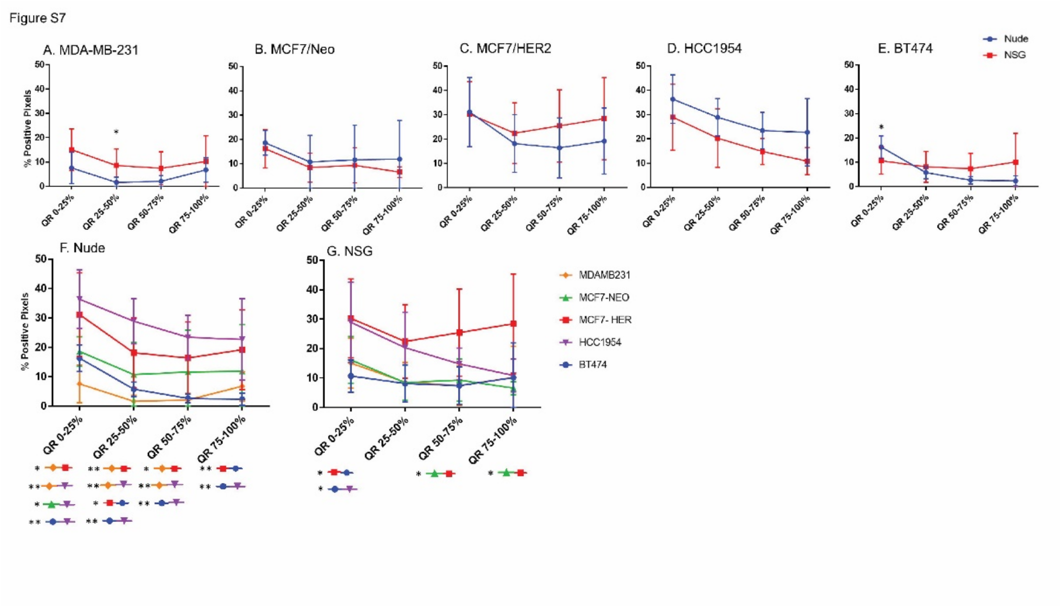

Figure S7: Nude mice tumors showed significant difference in collagen content by Masson's trichrome staining. (Mann Whitney - *p<0.05; **p<0.01).

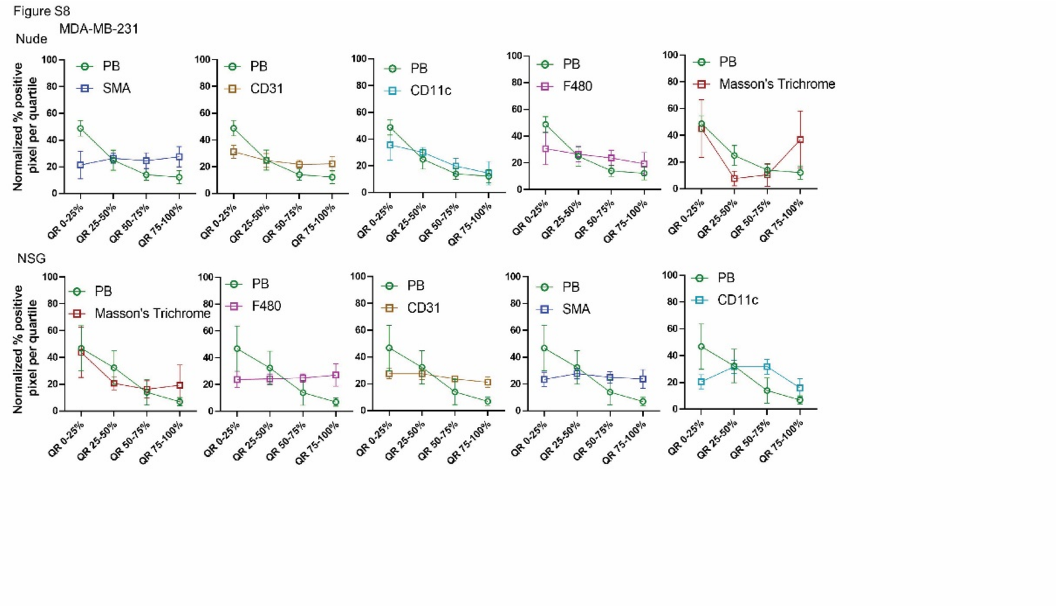

Figure S8: BH nanoparticle distribution positively correlates most with fibroblasts (SMA), followed by blood vessels (CD31) in MDA-MB-231 tumors grown in nude mice. In NSG mice tumors, nanoparticle distribution co-related most with Masson's Trichrome for collagen followed by F4/80 for macrophages.

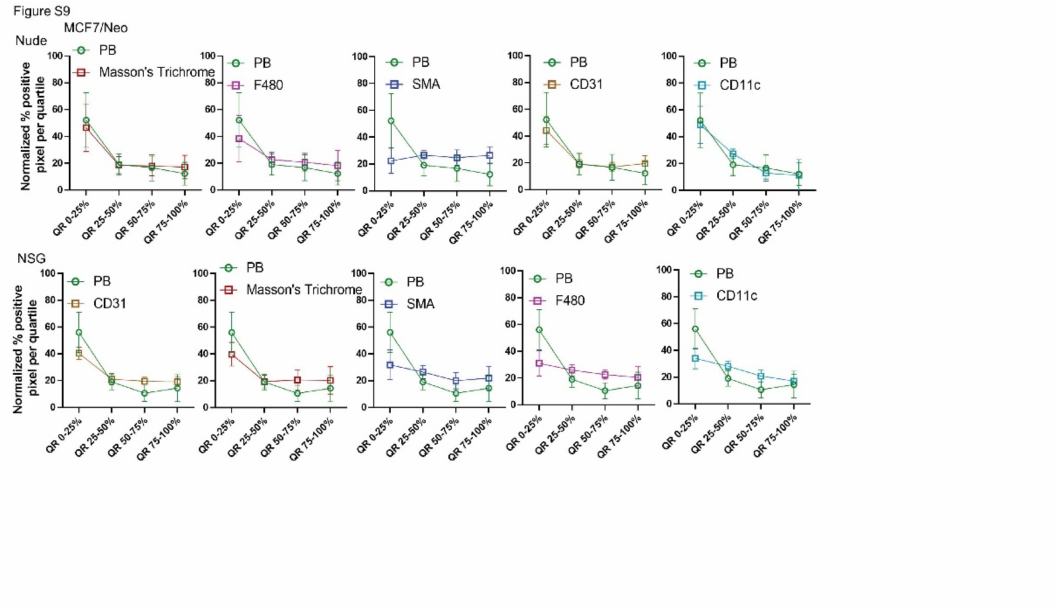

Figure S9: In MCF7/neo tumors there was a positive correlation between BH nanoparticle distribution and each cell type analyzed. Collagen (Masson's Trichrome) and macrophages (F4/80) correlated most followed by fibroblasts (SMA),blood vessels (CD31) and proinflammatory and dendritic cells (CD11c) in in nude mice. In NSG mice tumors, nanoparticle distribution co-related most with fibroblasts (SMA), collagen (Masson's Trichrome), blood vessels (CD31), macrophages (F4/80) and proinflammatory and dendritic cells (CD11c).

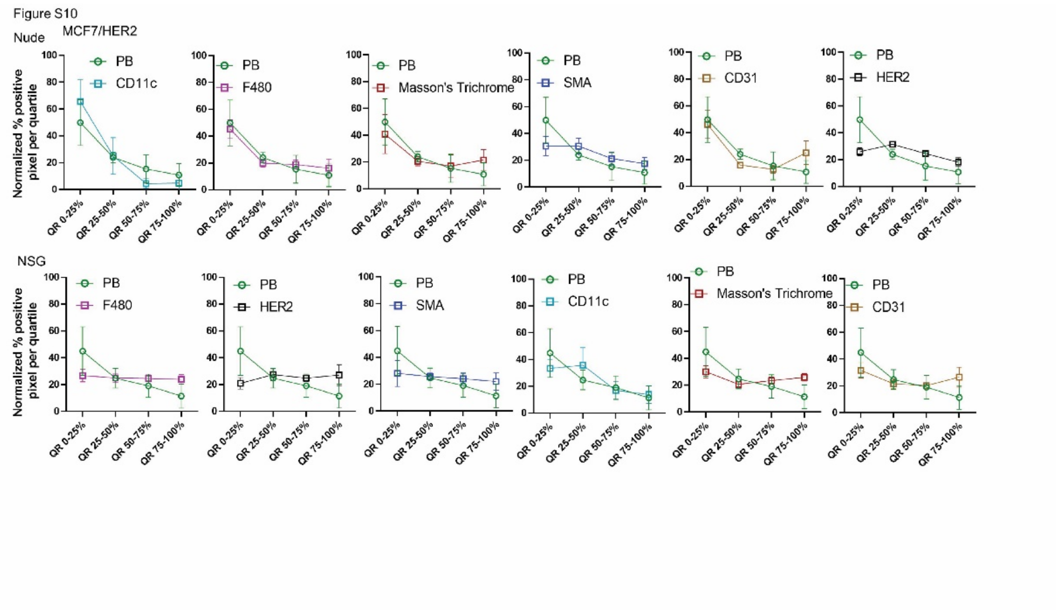

Figure S10: HER2 overexpressing MCF7/HER2 tumors showed highly positive correlation between BH nanoparticle distribution and CD11c cells (proinflammatory and dendritic cells) and F4/80 (macrophages) cells followed by Masson's Trichrome (collagen) stained areas. In NSG mice, the distribution was mostly correlated with macrophages (F4/80). A slight positive correlation was found with HER2+ve tumor cell distribution.

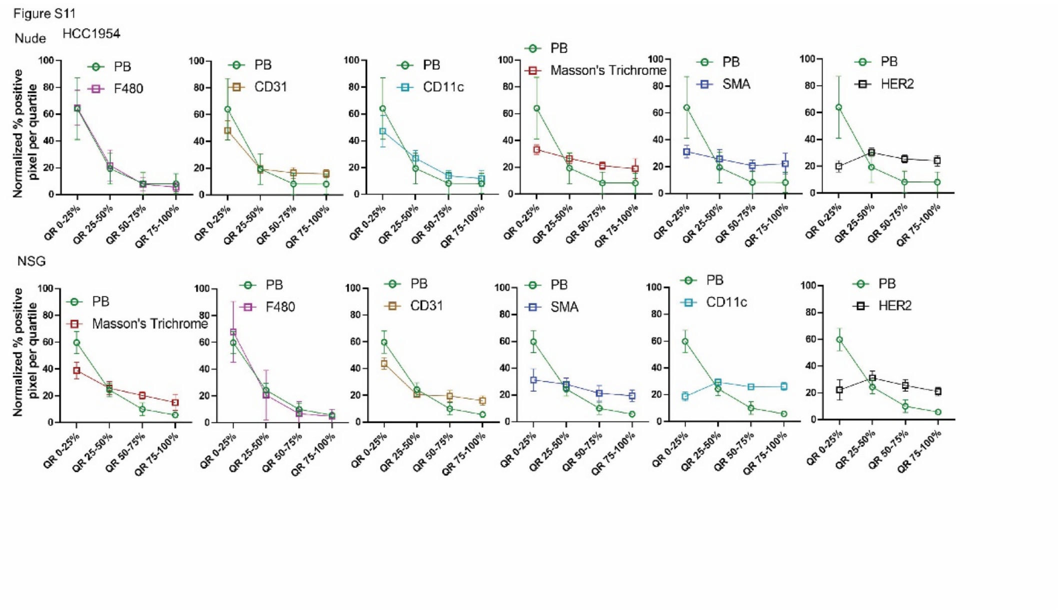

Figure S11: Inherently HER2 overexpressing HCC1954 tumors grown in nude and NSG mice also showed positive correlation between BH nanoparticle distribution and immune and stromal cells with no correlation with HER2+ve tumor cells. In nude mice, the correlation was highest with macrophages (F4/80) followed by CD31 (blood vessels), CD11c (proinflammatory and dendritic cells), Masson's Trichrome staining (collagen) and SMA cells (fibroblasts). In NSG mice it correlated most with collagen areas (Masson's Trichrome) followed by macrophages (F4/80) and blood vessels (CD31).

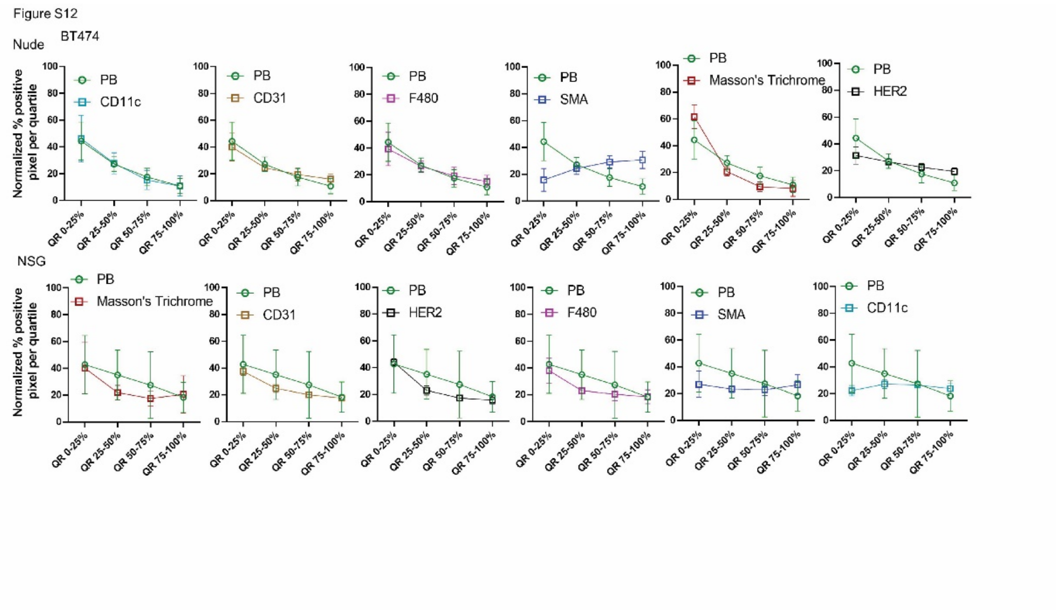

Figure S12: Another HER2 overexpressing BT474 tumors grown in nude mice also showed positive correlation between BH nanoparticle distribution and each cell type tested including HER2+ve tumor cells. Whereas in NSG mice growing same tumors, the correlation was highest with Masson's Trichrome staining (collagen) followed by CD31 (blood vessels) and tumor cells (HER2).
